# A taxonomic revision of the New World genus *Oropodes* Casey (Coleoptera, Staphylinidae, Pselaphinae)

**DOI:** 10.3897/zookeys.147.2072

**Published:** 2011-11-16

**Authors:** Donald S. Chandler, Michael S. Caterino

**Affiliations:** 1 Department of Biological Sciences, University of New Hampshire, Durham, NH 03824; 2 Curator of Entomology, Santa Barbara Museum of Natural History, Santa Barbara, CA 93105-2998

**Keywords:** Coleoptera, Staphylinidae, Pselaphinae, Californian, revision

## Abstract

The genus *Oropodes* is characterized and revised with 18 species being treated. Members of this genus are found in temperate forests to desert brush lands from Oregon to Baja California, but are associated primarily with dry forests and shrub lands of California. Keys to males and females, where known, are provided. Seven species are redescribed: *Oropodes arcaps* (California), *Oropodes dybasi* (Oregon), *Oropodes ishii* (California), *Oropodes nuclere* (California), *Oropodes orbiceps* (California), *Oropodes rumseyensis* (California), *Oropodes yollabolly* (California). The name *Oropodes raffrayi* (California) is raised from synonymy and the species is redescribed, NEW STATUS. Ten new species are described: *Oropodes aalbui* (California), *Oropodes bellorum* (California), *Oropodes casson* (California), *Oropodes chumash* (California), *Oropodes esselen* (California), *Oropodes hardyi* (California), *Oropodes serrano* (California), *Oropodes tataviam* (California), *Oropodes tongva* (California), and *Oropodes tipai* (Baja California, Mexico), NEW SPECIES. These species are placed into three species groups: the arcaps-group, the orbiceps-group, and the raffrayi-group. Data for a new record of *Euplecterga fideli* are given, and a list of the unassociated *Oropodes* females with distinctive genitalia is included with their label data.

## Dedication

This paper is presented in honor of Ross and Joyce Bell. The first author has always looked forward to the hospitality, company, and discussion with them on topics treating Coleopterology and ecology of New England, that made a stop in Burlington such a pleasure.

## Introduction

Thomas Casey (1894) described the new genus and species, *Oropodes orbiceps*, for a single specimen taken in Los Angeles County, California, that had “a facies which is somewhat intermediate between *Euplectus* and *Oropus*.” Later in the same year Brendel (1894) described *Euplectus raffrayi* from “California,” a name that was subsequently listed as a junior synonym of *Oropodes orbiceps* by Raffray (1904) without discussion. Fall (1901) provided the first and for many years the only comment on the biology of this group under the name *Euplectus orbiceps*. He stated that this species was uncommon and had been taken by sifting leaf litter at Pomona, Pasadena, and near Ojai, localities in or west of the northern portion of the Los Angeles Basin. The lack of any further published comments on this group, plus their apparent rarity, reinforced the impression that *Oropodes* was restricted to the general area of Los Angeles - a unique distribution for a Californian genus of Pselaphinae. In western North America pselaphine genera are most diverse in the woodlands and forests of northern and central California to the Pacific Northwest. From 1954 into the 1960’s Robert O. Schuster and colleagues commenced serious sampling of leaf litters in northern California. One product of these efforts was a revision of this genus by Grigarick and Schuster (1976), who described four new species from northern California and Oregon. Two additional species from northern California were added by (Chandler (1983, 2003), to bring the total to seven species.

While best known from the Los Angeles Basin, *Oropodes orbiceps* has been recorded as being widespread in California, with single records from Mt. Diablo (Contra Costa County; Grigarick and Schuster 1976) and Tehama County (Chandler 2003). However, the Tehama County record is now known to be incorrect, and the record for Mt. Diablo is highly suspect - unfortunately the specimen could not be located. All other known species appeared to have more limited distributions, a typical pattern for Californian pselaphines. A recent spate of collecting by the second author and colleagues, that focused on the inadequately sampled Transverse Range area of southern California, has produced a number of undescribed species from Monterey to San Bernardino Counties. These, plus additional material from other Pacific state localities and reinstatement of *Oropodes raffrayi* as a valid species, have now brought the total for the genus to 18 species, with 10 of these species being undescribed. All species described here are based on males, with six of these lacking associated females. Females have their own distinctive genitalia, and we have seen seven distinct forms that we cannot reliably associate with males. These females are not described here as species, since the patterns of distribution for species are so inadequately known, and the ranges of species based solely on males are typically known from single collections or specimens. The process of focused collecting in different areas of California has consistently generated undescribed species, which strongly suggests that additional collecting from unsampled or undersampled areas from Oregon to Baja California will produce additional species.

## Methods

Measurements are in millimeters. Length is taken from the tip of the labrum to the abdominal apex. All specimens were initially dried and mounted on points. The male and female sexual characters were examined after clipping off the tip of the point with the specimen and placing into hot 10% KOH for 15-20 minutes for maceration. For some females the specimen were dropped into hot water for 15 minutes, placed in a small dish with 70% ethanol, the apical sternites removed, and then similarly macerated. Once cleared the specimens or abdomens were placed in 70% ethanol for a minute, and then positioned in a drop of glycerin on a microscope slide. The genitalia were extracted, and the parts covered by a cover slip, supported laterally by cover slips glued to the slide. The slide was then placed on the stage of a Wild M11 compound microscope, the desired parts manipulated until they were in the correct position, and the features sketched using a camera lucida. Additional features were checked on dried specimens using a Wild M5 APO. After a preliminary sketch had been polished, features were checked again using a slide mount, and the various parts measured. Disarticulated specimens are stored in glycerin placed at the bottom of microvials and pinned through the stopper beneath the label data for the specimens.

Holotypes were examined only for the two oldest species (*Oropodes orbiceps* Casey and *Oropodes raffrayi* Brendel) whose identities were uncertain. Paratypes from series of the other described species were available, and the illustrations of the holotypes in the revision of Grigarick and Schuster (1976) allows precise determination of the species they described. Label data for these types begin and end with “//”, and individual labels are separated by “/”. Brackets are used when additional information is added to the type data. When habitat data were lacking for the undescribed species, when possible the original collector was contacted to obtain information on the vegetation typical of the collection area, or the terrain was checked using Google™ Earth.

Members of *Oropodes* have been taken using several different collecting techniques. Specimens may be taken by sifting/Berlesing a variety of leaf litters or root mats, and this has been particularly productive for species from Oregon and California north of the San Francisco Bay that were taken in series from redwood, Douglas-fir, or big-leaf maple leaf litters. Other techniques that have been profitable, particularly for a few species from southern California, are use of light traps and flight intercept traps, with the only known specimen of one species (*Oropodes aalbui* n. sp.) found in a pitfall trap.

## Material

This study was based on the study of 310 specimens. The following codens indicate the collections from which specimens have been borrowed or are deposited. Names of the curators of these collections are in parentheses.

ANSP Academy of Natural Sciences, Philadelphia, PA. (Jason D. Weintraub).


CASC California Academy of Sciences Collection, San Francisco, CA. (David H. Kavanaugh).


CSCA California State Collection of Arthropods, Sacramento, CA. (Charles L. Bellamy).


CNCI Canadian National Collection of Insects, Agriculture Canada, Ottawa, ON. (Yves Bousquet).


DSC Donald S. Chandler collection, University of New Hampshire, Durham, NH.


EMUS Essig Museum of Entomology, University of California, Berkeley, CA. (Cheryl B. Barr).


FMNH Field Museum of Natural History, Chicago, IL. (Margaret K. Thayer).


MCZC Museum of Comparative Zoology, Harvard University, Cambridge, MA. (Philip D. Perkins).


MNHN Museum National d’Histoire Naturelle, Paris, France. (Azadeh Taghavian).


SBMN Santa Barbara Museum of Natural History, Santa Barbara, CA.


UCDC University of California, Davis, CA. (Steven L. Heydon).


UCRC University of California, Riverside, CA. (Douglas Yanega).


USNM U.S. National Museum, Washington, DC. (Gary F. Hevel).


## Taxonomy

### Genus Oropodes

Casey, 1894

http://species-id.net/wiki/Oropodes

Oropodes Casey, 1894: 453. Type species: *Oropodes orbiceps* Casey, by monotypy. Raffray 1898: 211 (key), 246; 1904: 526 (key), 565; 1908: 41 (key), 81; 1911: 27. Bowman 1934: 8 (key), 25. Arnett 1960: 315 (key), 321. Park and Wagner 1962: 15. Grigarick and Schuster 1976:97 (revision); 1980: 27, pl. 29. Newton and Chandler 1989: 23 (catalog). Chandler 1990: 1187 (key); 1997: 15 (catalog); 2000: 290 (key), 347; 2001: 107; 2002: 57 (key), 79.

#### Description.

**Figure 1.**
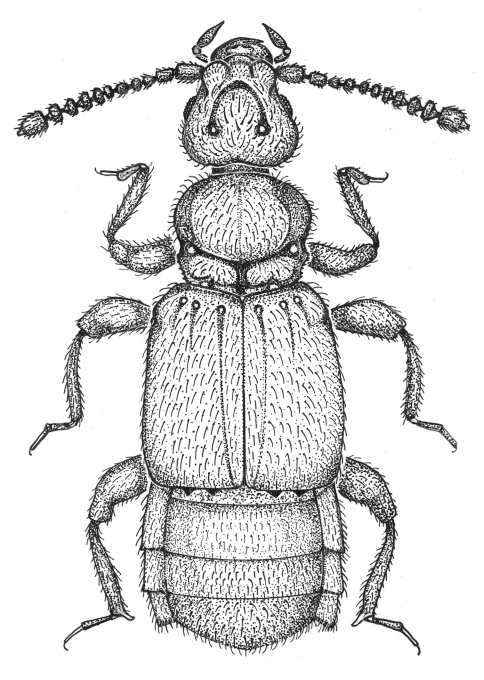
Habitus of *Oropodes chumash*. Body length equals 1.9 mm. Scale line equals 0.1 mm.

Dorsal habitus as in Figure 1. Length 1.68-2.40. Body light yellow-brown to brown. Head trapezoidal, narrowing apically, slightly narrower than pronotum, base nearly straight, vertexal foveae nude, connected by an inverted U-shaped sulcus; with prominent rounded antennal tubercles, lacking interantennal ridge; with 11 antennomeres, IX-XI forming loose club, X symmetrical and usually wider than IX, antennomeres V and VII usually slightly larger than adjacent antennomeres; head venter with short sparse setae angled anteriorly, with faint median gular suture, single gular fovea at head base, with gular boss at base of mentum.

Pronotal disc with shallow median longitudinal sulcus extending to base, lateral longitudinal sulci faint to indistinct; antebasal sulcus deep, forming a broad V between nude lateral antebasal foveae, lacking median antebasal fovea; lateral margins convergent basally, carinate in basal half, slightly constricted adjacent to lateral foveae, margins denticulate from constriction to base; base with lateral margins polished, forming two vague oval impressions to each side. Prosternum convex, with lateral prosternal carinae extending from anterolateral corner of procoxae obliquely dorsally to cervix; lateral procoxal foveae present.

Elytra with 3 basal foveae, short discal stria extending from lateral foveae no more than one-third elytral length; with subhumeral fovea; with apicolateral cleft. Median mesosternal fovea broadly forked from single opening, lateral mesosternal foveae broadly forked, anterior fork small and isolated; lateral mesocoxal foveae present; lacking metasternal foveae. Hindwings fully developed.

Abdomen with visible tergites 1-3 subequal in length, 4 slightly longer; 1 with basolateral foveae, deep depression between foveae nude, anterior face of depression with two large blunt teeth facing posteriorly; tergites lacking discal carinae at base. Metacoxal cavities angularly prolonged posteriorly near mesal margin; first visible ventrite usually with short carinae extending posteriorly from apex of angulation to ventrite apex, medial area between carinae flat or nearly so; second ventrite with inner and outer basolateral foveae in setose basolateral sulci. Profemora lacking ventral sensory pits or impression; tibiae with apical ctenidia of few to several spines on anterior and posterior margins.

Males with third ventrite bearing median anteriorly-directed lamina. Aedeagus with dorsal diaphragm; parameres asymmetric, flattened and fused at base.

#### Biology.

 Specimens of *Oropodes* have been taken in the widest array of habitats possible for pselaphines in California, ranging from redwood forests, high elevation Douglas-fir forests, gallery forests in grassland areas, dry chaparral/ pine forests, foothill woodlands, grass roots, an urban residential area, and from a cave in an area of high desert scrub. Many of the species have been found at sites dominated by dry pine/oak forests mixed with brush or chaparral. Use of passive traps (light traps, flight intercept traps) in southern half of California have been the most productive techniques in collecting species, as species have been difficult to find by the usual procedure of berlesing leaf litter or root mat samples.

Adults seasonality exhibits two broad patterns: the species from northern California, where rain is received during the summer, have collection records from months throughout the year. Those species occurring where there are long periods without rain, particularly those from southern California, appear in late autumn and early winter, and may be taken until June when the California summer is well under way.

#### Relationships.

*Oropodes* is certainly close to *Euplecterga* Park & Wagner, 1962, being effectively the sister-group in view of the latter genus being initially described as a subgenus of *Oropodes*. They are similar in habitus, sharing the somewhat flattened body, the slightly enlarged seventh antennomere, the shallow median sulcus of the pronotum, the abdominal tergites subequal in length, and with the males possessing a median lamina on the third ventrite and lacking sensory pits on the profemora. The two genera are separated by the presence of promesocoxal foveae, the lack of inner basolateral foveae on the second ventrite, the first ventrite lacking longitudinal carinae arising at the posteromedial angulations of the metacoxal cavities, and the asymmetrical male eleventh antennomere for *Euplecterga*, while *Oropodes* lacks promesocoxal foveae, both inner and outer basolateral foveae are present on the second ventrite, nearly all species have distinct longitudinal carinae arising at the posteromedial angulations of the metacoxal cavities that extend posteriorly to the apex of the first ventrite, and the male eleventh antennomere is symmetrical. The two genera are indeed similar in overall appearance, and a single specimen from the San Francisco Bay area was in the process of being treated as an undescribed species of *Oropodes* before the first author fortunately realized it was the second known specimen of *Euplecterga fideli* Grigarick & Schuster (1976). The male holotype of *Euplecterga fideli* was collected in Santa Cruz County, 9 mi NE of Soquel, while the newly discovered male has the data: San Mateo County, Lake Pilarcitos, III-20-1965, C.W. & L.B. O’Brien, shore debris (CNCI)].

The mesosternal foveal pattern places these two genera clearly in the subtribe Trichonychina (Chandler 2001: 107). Grigarick and Schuster (1980: 25) produced a tree for the twelve North and Central American genera in their “Group B” (= Trichonychina) indicating two major clusters of genera based on a few characters, with a group holding the genera *Bontomtes* Grigarick & Schuster and *Foveoscapha* Park & Wagner being placed adjacent to *Oropodes* in one major cluster, and *Euplecterga* associated with *Tetrascapha* Schuster & Marsh in the other major cluster. The subequal lengths of the abdominal tergites, lack of lateral metasternal foveae, and the somewhat flattened body are features found only in *Oropodes* and *Euplecterga* amongst these genera (metasternal foveae lacking in *Tetrascapha*), while the sulcate pronotal disc is shared with *Bontomtes* and *Foveoscapha*. These last two genera are more robust in appearance and the lateral metasternal foveae are present.

Within the genus the species are placed into three preliminary species-groups: the arcaps-group (4 species), the orbiceps-group (4 species), and the raffrayi-group (10 species). In the taxonomic section the species-groups are treated alphabetically, with the included species of each group similarly treated alphabetically.

#### Keys to species of *Oropodes*.

Males may be identified using the secondary sexual characters of the legs, abdominal lamina, apical sclerites, and genitalia, while females have valuable characters on the apical sclerites of the abdomen and the internal genitalia. Due to the possibility of encountering undescribed species we advise that the genitalia be extracted, macerated, and viewed. Also, the leg characters of the male tibiae may be obscured by dense setae, and are difficult to see unless placed on a slide in a glycerin mount. Caution is urged when identifying isolated females. Females are not known for six of the species described here, and we have seen seven female morphospecies with distinctive genitalia that lack associated males. Males always have a recurved lamina following an impression at the middle portion of the third ventrite, and at least the second ventrite is medially impressed. Females lack the lamina on the third ventrite, which together with the second ventrite are both convexly and evenly rounded.

##### Key to males.

**Table:** 

1	Profemora lacking tooth or tubercle on ventral margin near base; second ventrite with posterior margin straight (Fig. 2C); eyes relatively small, with 12-40 facets; north of the San Francisco Bay Area to Oregon	2
–	Profemora with tooth or tubercle on ventral margin near base (Fig. 6B); second ventrite often with pair of acute teeth or rounded lobes as wide apart as width of impression on third ventrite (Fig. 13C); eyes larger, with about 45-70 facets, usually ranging from about 60-70; northern California to Baja California	5
2(1)	Metatrochanters with short acute spine on posterior margin (Fig. 3G); lamina of third ventrite with anterior margin broadly and slightly concave in ventral view (Fig. 3C); Oregon	2. *Oropodes dybasi* Grigarick & Schuster
–	Metatrochanters smoothly rounded on posterior margin; lamina of third ventrite with anterior margin slightly to strongly convex (Fig. 4C); California	3
3(2)	Protibiae with blunt angulation at middle of mesal margin; mesotibiae with blunt preapical tubercle (Fig. 4B); sixth ventrite with setose area strongly and broadly constricted at middle (Fig. 4D); Butte and Tehama Counties	3. *Oropodes ishii* Chandler
–	Protibiae with mesal margin smooth, lacking angulations; mesotibiae lacking preapical tubercles (Fig. 2B); sixth ventrite with margins of setose area constricted or parallel (Fig. 2D, 5D)	4
4(3)	Sixth ventrite with margins of setose area strongly and broadly constricted at middle (Fig. 2D); aedeagus with sinuate narrow spine in internal sac (Fig. 2A); Marin to Mendocino Counties	1. *Oropodes arcaps* Grigarick & Schuster
–	Sixth ventrite with margins of setose area evenly narrowing to middle (Fig. 5D); aedeagus with thick, slightly curved spine in internal sac (Fig. 5A); Tehama County	4. *Oropodes yollabolly* Chandler
5(1)	Second ventrite with posterior margin straight, lacking pair of distinct teeth or rounded lobes (Fig. 6C); species found in southern California south of the Tehachapi Mountains to Baja California	6
–	Second ventrite with pair of teeth or rounded lobes projecting at point even with lateral margins of impression of third ventrite (Fig. 13C); throughout California	9
6(5)	Lamina arising at apex of third ventrite (Fig. 8C); protibiae subangulate on mesal margin past middle (Fig. 8B)	7
–	Lamina arising at point about two-thirds from anterior margin of third ventrite (Fig. 7C); protibiae with mesal margin smoothly and barely curved (Fig. 7B)	8
7(6)	Mesotibiae with straight apical spur on mesal margin (Fig. 6B); aedeagus with left paramere sinuate at apex (Fig. 6A); Santa Barbara and Los Angeles Counties	5. *Oropodes orbiceps* Casey
–	Mesotibiae lacking apical spur, with two widely spaced preapical tubercles on mesal margin (Fig. 8B); aedeagus with left paramere truncate at apex (Fig. 8A); Los Angeles County	7. *Oropodes tataviam* Chandler & Caterino, sp. n.
8(6)	Mesotibiae with two close preapical tubercles (Fig. 7B); aedeagus with left paramere strongly sinuate at apex (Fig. 7A); San Bernardino County	6. *Oropodes serrano* Chandler & Caterino, sp. n.
–	Mesotibiae with single small preapical tubercle (Fig. 9B); aedeagus with left paramere truncate at apex (Fig. 9A); northern Baja California	8. *Oropodes tipai* Chandler & Caterino, sp. n.
9(5)	Lamina of third ventrite originating near middle; projections at posterior margin of second ventrite more rounded (Fig. 13C)	10
–	Lamina of third ventrite originating close to posterior margin; projections at posterior margin of second ventrite typically more acute (Fig. 19C)	12
10(9)	Width of lamina of third ventrite about one-fifth ventrite width (Fig. 12C), lamina raised at about 40°; Tulare County	11. *Oropodes casson* Chandler & Caterino, sp. n.
–	Width of lamina of third ventrite close to one-third ventrite width (Fig. 13C), lamina raised at about 25°	11
11(10)	Aedeagus with apex of left paramere broad and sinuate (Fig. 13A); Santa Barbara and Los Angeles Counties	12. *Oropodes chumash* Chandler & Caterino, sp. n.
–	Aedeagus with apex of left paramere narrower and bluntly pointed (Fig. 10A); San Bernardino County	9. *Oropodes aalbui* Chandler & Caterino, sp. n.
12(9)	Protibiae with large blunt tubercle on mesal margin in basal half, broadly emarginate to small tubercle in apical half; ventral tooth of profemora large and slanted (Fig. 19B); Los Angeles County	18. *Oropodes tongva* Chandler & Caterino, sp. n.
–	Protibiae bluntly angulate near middle to smoothly curved; profemoral tooth more erect (Fig. 19B)	13
13(12)	Protrochanter with large truncate projection on ventral margin (Fig. 11B); metatrochanters with ventral margin angulate; lamina of third ventrite with apex straight (Fig. 11C); Calaveras County	10. *Oropodes bellorum* Chandler & Caterino, sp. n.
–	Pro- and metatrochanters with ventral margins smoothly curved, not modified; lamina of third ventrites with apex concave (Fig. 15C)	14
14(13)	Profemoral tooth as long as wide or longer; mesotibiae with two widely spaced preapical tubercles on mesal margin, lacking apical spur (Fig. 14B); left paramere with apex subtruncate, projecting well beyond apex of right paramere (Fig. 14A)	15
–	Profemoral tooth half as long as wide; mesotibiae with long apical spur on mesal margin, lacking preapical tubercles (Fig. 18B); both parameres about same length, armature of internal sac often obscuring parameres when viewed dorsally	16
15(14)	Male metasternum with median longitudinal sulcus; lamina of third ventrite with apical portion horizontal; apex of left paramere narrowly subtruncate (Fig. 15A); body 2.3-2.4 mm long; Sacramento County	14. *Oropodes hardyi* Chandler & Caterino, sp. n.
–	Male metasternum convex (only species with this character); lamina of third ventrite angled at about 30°; apex of left paramere broadly subtruncate (Fig. 14A); body 1.7-2.1 mm long; Monterey and San Luis Obispo Counties	13. *Oropodes esselen* Chandler & Caterino, sp. n.
16(14)	Lamina of third ventrite horizontal at apex; rod in internal sac with multiple teeth in apical portion (Fig. 17A); “California” 16. *Oropodes raffrayi* (Brendel)
–	Lamina of third ventrite angled at about 30°; rod of internal sac with 1-2 apical spines (Fig. 18A)	17
17(16)	Aedeagus with rod of internal sac with two apical spines (Fig. 16A); Napa and Lake Counties	15. *Oropodes nuclere* Grigarick & Schuster
–	Aedeagus with rod of internal sac with single apical point (Fig. 18A); Marin to Tehama Counties	17. *Oropodes rumseyensis* Grigarick & Schuster

##### Key to females.

**Table:** 

1	Fifth ventrite with anterior and posterior margins of setose area parallel (Fig. 2F); genitalia with membranous lobe symmetrical or nearly so (Fig. 2E)	2
–	Fifth ventrite with setose area sharply constricted or divided at middle (Fig. 18F); membranous lobe of genitalia symmetrical or nearly so to strongly asymmetrical	5
2(1)	Posterior margin of fifth ventrite with broad truncate tab at middle (Fig. 3F); membranous lobe of genitalia with two arcuate rods (Fig. 3E)	2. *Oropodes dybasi* Grigarick & Schuster
–	Posterior margin of fifth ventrite evenly curved to nearly straight, lacking median projection (Fig. 2F); membranous lobe of genitalia different	3
3(2)	Membranous lobe of genitalia with two recurved spines, each with median projection (Fig. 2E)	1. *Oropodes arcaps* Grigarick & Schuster
–	Membranous lobe of genitalia lacking obvious sclerites (Fig. 4E) 4
4(3)	Found in the northern Sierra Nevada (Fig. 4)	3. *Oropodes ishii* Chandler
–	Found in the northern Coast Ranges of California (Fig. 5)	4. *Oropodes yollabolly* Chandler
5(1)	Membranous lobe of genitalia strongly asymmetrical (Fig. 18E)	5
–	Membranous lobe of genitalia symmetric or nearly so, internal sclerites may vary in size (Fig. 13E)	7
6(5)	Fifth ventrite with setose area narrowly closed at middle by projection from anterior margin (Fig. 18F); fifth tergite with blunt median tubercle at apex	17. *Oropodes rumseyensis* Grigarick & Schuster
–	Fifth ventrite with setose area broadly divided at middle by bar (Fig. 16F); fifth tergite with shallow median impression at apex	15. *Oropodes nuclere* Grigarick & Schuster
7(5)	Fifth ventrite with setose area narrowly closed or nearly so by projection from anterior margin (Fig. 13F)	8
–	Fifth ventrite with setose area broadly divided by bar at middle (Fig. 15F)	10
8(7	Fifth tergite with two close teeth at apex (Fig. 8H)	7. *Oropodes tataviam* Chandler & Caterino, sp. n.
–	Fifth tergite apex rounded	9
9(8)	Metasternum with disc convex; genitalia with two narrow sclerotized rods in membranous lobe (Fig. 13E)	12. *Oropodes chumash* Chandler & Caterino, sp. n.
–	Metasternum with median longitudinal sulcus; membranous lobe of genitalia with broad plate apically notched (Fig. 19E)	18. *Oropodes tongva* Chandler & Caterino, sp. n.
10(7)	Membranous lobe of genitalia with broad plate bearing apical point (Fig. 14E)	13. *Oropodes esselen* Chandler & Caterino, sp. n.
–	Genitalia with sclerites of membranous lobe formed differently	11
11(10)	Genitalia with membranous lobe elongate, with two flattened elongate plates of variable width (Fig. 6E); fifth tergite with distinct notch at apex (Fig. 6H)	5. *Oropodes orbiceps* Casey
–	Genitalia with membranous lobe about as long as wide, with broad lightly sclerotized plate (Fig. 15E); fifth tergite lacking apical notch	14. *Oropodes hardyi* Chandler & Caterino, sp. n.

##### Unassociated female specimens with distinctive genitalia.

The unassociated females we have seen are listed here so that the specimens may be located by future workers. Five are represented by single specimens.

1) Fresno County: 9 mi E Coalinga, III-20/VI-4-1981, Gilbert & Andrews (CSCA, 2 specimens).

2) Fresno County: Sequoia National Forest, 3 mi W Cedar Grove, 4400’, V-14-1976, A. Newton & M. Thayer (FMNH).

3) Los Angeles County: Point Mugu State Park, Boney Mountain State Wilderness, 34.1354°N, 118.9524°W, V-3-2009, M.S. Caterino & K.J. Hopp, *Umbellularia*/*Platanus* litter (SBMN, 2 specimens).

4) Madera or Mariposa County: Ahwahnee, May, A. Fenyes Collection (CASC). [either Ahwahnee, town in Madera County, 37.3639°N, 119.7203°W; or Ahwahnee Lodge in Yosemite Valley (Mariposa County), 37.7458°N, 119.5742°W]

5) Santa Barbara County: LPNF [Los Padres National Forest], Oso Canyon, IV-28-2002, M. Caterino (SBMN).

6) Tehama County: 6 mi SE Manton, Soap Creek, 716 m, XII-4-1991, D. S. Chandler, sift willow/mixed litter by stream (DSC).

7) Tulare County: Ash Mountain Power Station, XI-23-1982, J.A. Halstead (CNCI).

##### The arcaps-group.

Included species: *Oropodes arcaps* Grigarick & Shuster, *Oropodes dybasi* Park & Wagner, *Oropodes ishii* Chandler*, and O. yollabolly* Chandler.

Diagnostic features: Eyes relatively small, 12-40 facets. Males lacking basal spine on venter of profemora; second ventrite with posterior margin smooth, lacking teeth or lobes. Females with transverse margins of setose area of fifth ventrite parallel; genitalia with membranous lobe symmetrical or nearly so.

**Map 1.**
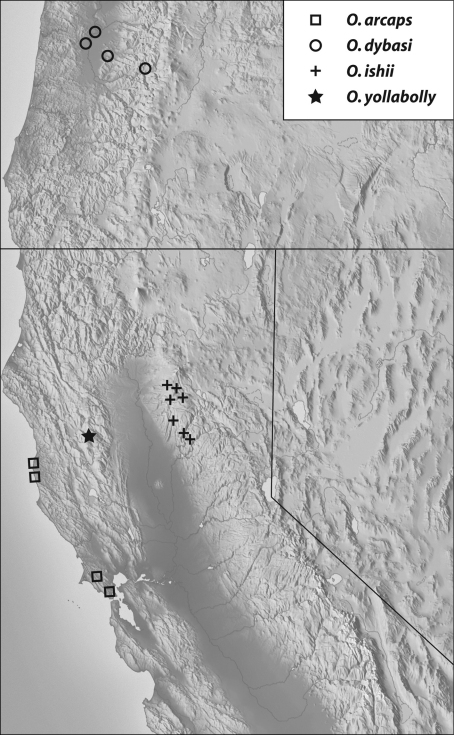
Records of thearcaps-group: *Oropodes arcaps*, *Oropodes dybasi*, *Oropodes ishii*, *Oropodes yollabolly*.

Geographical distribution: The range of this group extends from north of the San Francisco Bay area of California into Oregon (Map 1).

### 1. Oropodes arcaps

Grigarick & Schuster, 1976

http://species-id.net/wiki/Oropodes_arcaps

Figs 2 20 Map 1

Oropodes arcaps Grigarick & Schuster: 1976, 103; Chandler 1997: 15. Type locality: California, Mendocino County, Casper. Holotype male (UCDC).

#### Specimens examined.

44: CALIFORNIA: **Marin County:** 2.7 mi W Mt. Tamalpais, V-14-1988, VI-12-1988, I-8-1989, II-12-1989, S.T. O’Keefe, berlese duff *Sequoia sempervirans* (DSC); Forest Knolls, XII-13-1958, C.W. O’Brien (UCDC); Samuel P. Taylor State Park, II-3-1958, J.R. Helfer (UCDC); Samuel P. Taylor State Park, S entrance, XI-1-1958, R.O. Schuster & G.A. Marsh (UCDC). **Mendocino County:** 1 mi N Albion, VII-29-1978, D.S. Chandler, sift fern litter (DSC); Caspar, IX-30-1954, VII-29-1954, J.R. Helfer, (UCDC, 2 female paratypes).

#### Description.

Length 1.64-1.72. Body light orange-brown. Eyes with 17-20 facets at most localities, varying to around 35 facets for Mt. Tamalpais specimens. Antennomeres V and VII slightly larger than those adjacent, V-VIII obconical, IX narrower than X. Abdomen with first ventrite lacking carinae that extend from posteromedial angles of metacoxal cavities to ventrite apex.

#### Males:.

**Figure 2.**
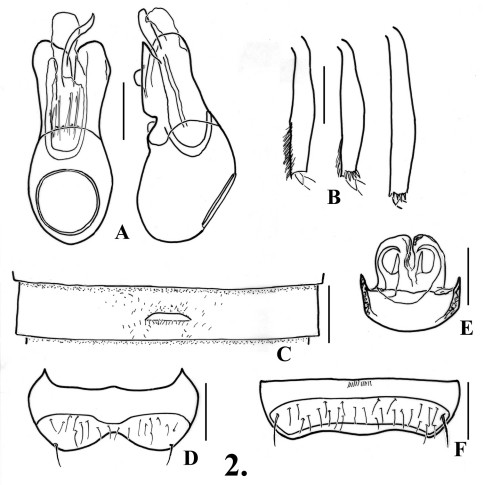
*Oropodes arcaps* **A** Dorsal and right lateral view of male genitalia **B** Posterior view of right male protibia, mesotibia, and metatibia **C** Ventral view of male third ventrite **D** Ventral view of male sixth ventrite **E** Dorsal view of female genitalia **F** Dorsal view of female fifth ventrite. Scale line equals 0.1 mm.

Metasternum with narrow median longitudinal sulcus. Legs (Fig. 2B): profemora simple; protibiae with small, slight angulation on mesal margin past middle; mesotibiae simple; metatibiae with very short apical spur on mesal margin. Abdomen (Figs 2C, 20) with second ventrite lightly flattened at middle fifth, posterior margin slightly humped but not projecting in line with lateral margins of lamina; third ventrite 0.58 wide, slightly concave in middle fourth anterior and posterior to lamina, lamina small, 0.08 wide, with apex broadly rounded to nearly straight at middle, lamina at about middle of ventrite, lamina flat, angled at about 20°; fourth and fifth ventrites flattened in middle fourth; sixth ventrite (Fig. 2D) flattened in middle third, with setose area broadly constricted to middle. Aedeagus (Fig. 2A) 0.42 long; parameres with apices broadly and irregularly rounded; internal sac with single long sinuate spine.

#### Females:.

Fifth tergite with setose area evenly convex. Fifth ventrite (Fig. 2F) with transverse margins of setose area parallel, posterior margin somewhat broadly but shallowly protruding at middle. Genitalia symmetric (Fig. 2E), median membranous lobe with thin arcuate sclerites meeting medially and with straight spine extending anteriorly from near their bases.

#### Collection notes.

Specimens were taken from redwood and fern leaf litters near or at the coast, indicating the strongest preference for wetter forests for a member of this genus.

#### Geographical distribution.

(Map 1): This species is found in coastal areas from Marin County in the San Francisco Bay area north to Mendocino County.

#### Comparisons and diagnostic notes.

The arcaps-group is based on this species, with the critical characters being a lack of a profemoral tooth, the second ventrite lacking a pair of projections on the posterior margin, and eyes comparatively small. This species shares the relatively small and anteriorly convex lamina that originates at the middle of the third ventrite with *Oropodes ishii* and *Oropodes yollabolly*, and is separated from these by a combination of the simple mesotibiae, the deeply constricted setose area of the sixth ventrite of the male, and curving forked spines of the female genitalia.

### 2. Oropodes dybasi

Grigarick & Schuster, 1976

http://species-id.net/wiki/Oropodes_dybasi

Figs. 3 23 Map 1

Oropodes dybasi Grigarick & Schuster, 1976: 101; Chandler 1997: 15. Type locality: Oregon, Benton County, Well’s Creek, 9 mi SW Philomath. Holotype male (FMNH).

#### Specimens examined.

23, all DSC except where indicated: OREGON: **Benton County**: 8 mi W Philomath, 1000’, V-12-1983, D.S. Chandler, sift forest leaf litter; McDonald Forest, Sulfur Springs Road, II-3-1973, G.L. Peters. **Lane County:** all H.J. Andrews Experimental Forest: Road 134, 1750’, Watershed 10, I-24-1981, G. Cassis, site 4, 1975 clearcut, *Pseudotsuga* litter; Road 1506, 2500’, L301A, III-12-1981, G. Cassis, site 9, 1954 clearcut, *Pseudotsuga*/*Rhododendron* litter; R.S. 7, 1450’, V-13-1983, D. S. Chandler, old growth, sift Douglas-fir leaf litter & moss; Road 130, 1750’, V-12-1984, D. S. Chandler, old growth, sift cedar litter by stream; Lookout Creek, 1950’, V-13-1984, D. S. Chandler, old growth, sift bigleaf maple litter; Road 1506, 3500’, V-14-1984, D. S. Chandler, old growth, berlese moss by stream; R.S. 20, 2250’, Road 134, V-12-1984, D. S. Chandler, old growth, sift Douglas-fir leaf litter; Road 350, 4050’, V-11-1984, D. S. Chandler, old growth, sift Douglas-fir leaf litter; Road 1506, 1900’, Lookout Creek, X-27-1982, G. L. Parsons, site 6, 1963 clearcut, litter; Road 1506, 1900’, Lookout Creek, VII-6-1982, X-27-1982, V-11-1983, X-26-1983, G. L. Parsons, site 7, 1954-55 clearcut, litter; Road 1506, 1800’, IX-6-1982, G. L. Parsons, site 31, 1950 clearcut, litter. **Linn County**: 6 mi W Crawfordsville, 1200’, IX-15-1973, E.M. Benedict, bigleaf maple duff (DSC, CNCI). Additional specimens from the H.J. Andrews Experimental Forest are in the Oregon State University Collection.

#### Description.

Length 1.88-2.04. Body light orange-brown. Eyes of both sexes with 20-32 facets. Antennomere V larger than those adjacent, V-VIII quite transverse, IX narrower than X. Pronotum with medial sulcus shallow. Abdomen with carinae of first ventrite extending from posteromesal margin of metacoxal cavities posteriorly to ventrite apex.

**Figure 3.**
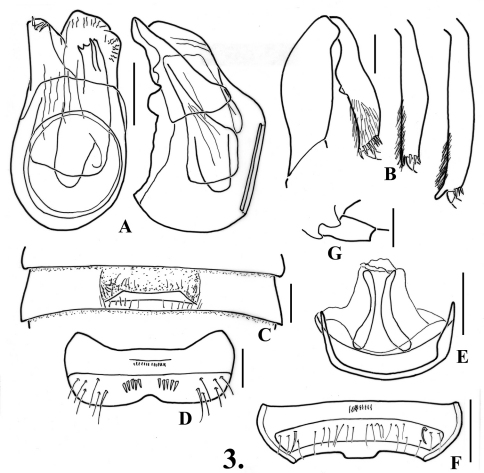
*Oropodes dybasi* **A** Dorsal and right lateral view of male genitalia **B** Posterior view of right male protibia, mesotibia, and metatibia **C** Ventral view of male third ventrite **D** Ventral view of male sixth ventrite **E** Dorsal view of female genitalia **F** Dorsal view of female fifth ventrite **G** Ventral view of male metatrochanter. Scale line equals 0.1 mm.

**Males:** Metasternum with broad median longitudinal impression. Legs (Fig. 3B): profemora not modified, protibiae angulate on inner margin at point one-third distance from base swollen from there to apex, inner margin broadly concave from angulation to apex; mesotibiae with small apical spine on mesal margin; metatrochanters with acute spine on ventral margin (Fig. 3G), metatibiae with spur at apex on mesal margin. Abdomen (Figs 3C, 23) with second-third ventrites impressed in middle third; third ventrite 0.60 wide, with lamina broad and thin, 0.23 wide, apex slightly and broadly emarginate on anterior margin, erect lamina arising just anterior to ventrite apex, strongly projecting and curved only near apex; fourth ventrite broadly impressed in middle two-fifths; fifth-sixth ventrites flattened in middle two-fifths, sixth ventrite (Fig. 3D) with oblique row of several thick setae to each side. Aedeagus (Fig. 3A) 0.34 long; left paramere broader than right, both with apices bluntly angulate; internal sac with armature, but form indistinct.

**Females:** Metatrochanter bluntly angulate on ventral margin near apex. Fifth tergite with setose area evenly convex; fifth ventrite (Fig. 3F) with margins of setose area parallel, apex with broad subrectangular tab at middle. Genitalia (Fig. 3E) with median lobe symmetrical, with 2 elongate curved rods in membranous lobe.

#### Collection notes.

During intensive sampling in 1981 and 1984 at the H.J. Andrews Experimental Forest in west-central Oregon, this species was taken most commonly in old growth Douglas-fir sites and from a 30 year-old clearcut regrowth, with only four specimens taken at sites cut more recently. In the Experimental Forest it has been found primarily in Douglas-fir leaf litter at low elevations. Most of the specimens were produced in the fall, winter, and spring, and seem to prefer drier sites. Most of this information is from unpublished data based on studies in the H.J. Andrews Experimental Forest by students of J. D. Lattin, and by the first author.

#### Geographical distribution.

(Map 1): This is the only species known from Oregon, and has been taken from forests at low to intermediate elevations on both sides of the Willamette Valley in west-central Oregon.

#### Comparisons and diagnostic notes.

Placed as a member of the *arcaps*-group, but the most distinctive member of the group. The male protibiae are angulate on the mesal margin and enlarged in the apical portion, the mesotibiae lack tubercles on the mesal margin, the metatrochanters have an acute spine on the mesal margin, the sixth ventrite has the setose area slightly constricted at the middle, and with short rows of thickened setae to each side of the midline. The female sixth ventrite has a broad tab on the apical margin, and there are two curved rods in the membranous lobe of the female genitalia. It is the only member of this group where the lamina of the third ventrite arises near the posterior margin and has the anterior margin shallowly concave.

### 3 Oropodes ishii

Chandler, 1983

http://species-id.net/wiki/Oropodes_ishii

Figs 4 21. Map 1

Oropodes ishii Chandler, 1983: 224; Chandler 1997: 15; 2003: 583. Type locality: California, Butte County, 5 mi NE Forest Ranch. Holotype male (UCDC).

#### Specimens examined.

33 (all DSC): CALIFORNIA: **Butte County** (all are paratypes): 5 mi NE Forest Ranch, I-3-1980, D.S. Chandler, sift tanbark oak and maple litter; same data except I-25-1980, sift rotten wood; same data except XI-28-1979, sift litter along stream; Griffin Gulch, 3 mi NW Concow Reservoir, 2000’, IV-2-1981, D.S. Chandler, sift tanbark oak litter; Mountain House, V-28-1981, D.S. Chandler, sift litter around spring; West Branch Feather River, 1600’, IV-13-1981, D.S. Chandler, sift oak and grape litter. **Tehama County**: Highway 32, Deer Creek, 1097 m, V-3-1981, sift Douglas-fir litter (paratypes); 19 mi SE Paynes Creek, S Fork Antelope Creek Canyon, south side, Ponderosa Way, 1006 m, XI-13-1998, D. S. Chandler, sift dogwood, Douglas-fir, and bigleaf maple litter; 14.5 mi SE Paynes Creek, Middle Fork Antelope Creek, 945 m, Ponderosa Way, XI-9-1998, D. S. Chandler, bigleaf maple leaf litter; 5 mi W Mineral, 1295 m, XII-6-1987, D. S. Chandler, sift bigleaf maple leaf litter; 5 mi SE Manton, Bluff Springs, 762 m, XII-1-1987, D. S. Chandler, sift fern leaf litter on stream edge; same site, 777 m, XII-4-1991, D. S. Chandler, sift fern leaf litter, sift laurel leaf litter; 6 mi SE Manton, Soap Creek, 716 m, XII-4-1991, D. S. Chandler, sift willow/mixed leaf litter by stream.

#### Description.

Length 1.82-1.90 mm. Body light orange-brown. Antennomeres V and VII slightly larger than those adjacent, V-VIII obconical, IX narrower than X, X symmetrical. Pronotum with median longitudinal sulcus shallow. Abdomen with first ventrite bearing short carina at inner margins of metacoxal cavity that extend to ventrite apex.

**Figure 4.**
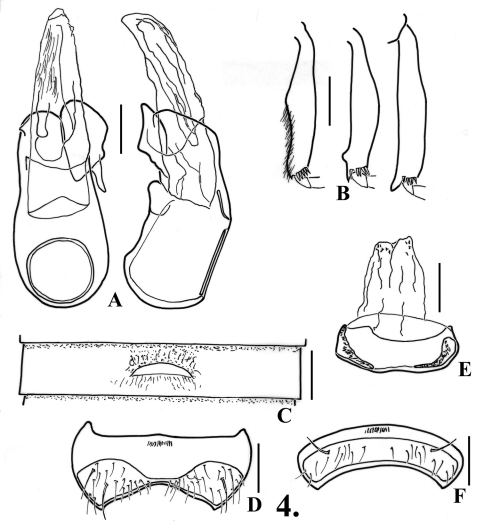
*Oropodes ishii* **A** Dorsal and right lateral view of male genitalia **B** Posterior view of right male protibia **C** Ventral view of male third ventrite **D** Ventral view of male sixth ventrite **E** Dorsal view of female genitalia **F** Dorsal view of female fifth ventrite. Scale line equals 0.1 mm.

**Males:** Eyes with 27–38 facets. Metasternum with shallow median longitudinal sulcus. Legs (Fig. 4B): profemora unmodified, protibiae broadly angulate at middle of mesal margin; mesotibiae with blunt preapical tubercle on mesal margin; metatibiae with large apical spur on mesal margin. Abdomen (Figs 4C, 21) with second ventrite broadly impressed in middle fifth; third ventrite 0.60 wide, broadly impressed in middle third, lamina 0.14 wide, apex of lamina broadly rounded, raised at about 30°, lamina arising just posterior to middle of ventrite; fourth ventrite broadly impressed in middle third; fifth ventrite flattened in middle third; sixth ventrite (Fig. 4D) flattened in middle third, setose area broadly constricted at middle by glabrous projection from anterior portion of ventrite. Aedeagus (Fig. 4A) 0.40 long; parameres broadly rounded at apex, right paramere projecting further, internal sac lacking large spines, often becoming extended during maceration of specimen in preparation for examination on a slide.

**Females:** Eyes with 12-32 facets. Fifth tergite with setose area convex; ventrites all broadly rounded at middle, lacking modifications, fifth ventrite (Fig. 4F) with setose area even in width. Genitalia (Fig. 4E) with median lobe elongate and membranous.

#### Collection notes.

Found in a variety of leaf litters, but the largest number of specimens were taken from Douglas-fir and Bigleaf maple leaf litters, and from litter by streams. It has been generally collected in the Ponderosa pine zone, but also has been occasionally collected at lower elevations in Butte County (Chandler, 2003).

#### Geographical distribution.

(Map 1): Known only from Butte and Tehama Counties, which are at the area of juncture between the north end of the Sierra Nevada and the southern portion of the Cascade Ranges (Mt. Lassen area).

#### Comparisons and diagnostic notes.

Placed as a member of the arcaps-group, and sharing with *Oropodes arcaps* and *Oropodes yollabolly* the anteriorly convex and medially placed lamina of the third ventrite. It is most similar to *Oropodes yollabolly* in the males sharing a preapical tubercle on the mesal margin of the mesotibiae and the metatibiae with an apical spur, and the elongate membranous lobe without any spines in the females. The males of these two species are easily separated by the medially constricted setose area of the sixth ventrite found in *Oropodes ishii*. The constricted ventrite is shared with *Oropodes arcaps*, whose males lack the preapical tubercle of the mesotibiae and the distinct apical spurs of the metatibiae, and the female genitalia has curved spines in the membranous lobe.

### 4 Oropodes yollabolly

Chandler, 2003

http://species-id.net/wiki/Oropodes_yollabolly

Figs 5 22 Map 1

Oropodes yollabolly Chandler, 2003: 584. Type locality: California: Tehama County, Grindstone Canyon, 7 mi W Log Springs, Mendocino National Forest. Holotype male (FMNH).

#### Specimens examined.

35, all DSC: CALIFORNIA: **Tehama County**: 32 Paratypes, Mendocino National Forest, 7 mi W Log Springs, Grindstone Canyon, 1326 m, XII-3-1991, D. S. Chandler, sift Douglas-fir litter by stream; 2 Paratypes, Mendocino National Forest, 6 mi W Log Springs, 1585 m, XI-29-1986, D. S. Chandler, sift maple, *Avens*, and oak leaf litter.

#### Description.

Length 1.88-2.20 mm. Body light orange-brown. Eyes with 17-23 facets. Antennomeres V and VII slightly larger than those adjacent, V-VIII obconical, IX narrower than X. Abdomen with carinae of first ventrite extending from posteromesal margins of metacoxal cavities posteriorly to ventrite apex.

**Figure 5.**
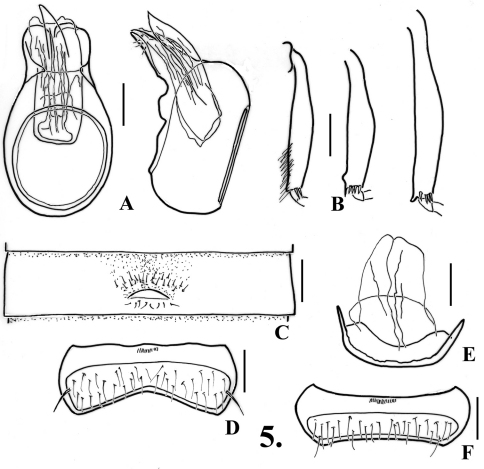
*Oropodes yollabolly* **A** Dorsal and right lateral view of male genitalia **B** Posterior view of right male protibia, mesotibia, and metatibia **C** Ventral view of male third ventrite **D** Ventral view of male sixth ventrite **E** Dorsal view of female genitalia **F** Dorsal view of female fifth ventrite. Scale line equals 0.1 mm.

**Males:** Metasternum with shallow median longitudinal impression. Legs (Fig. 5B): profemora simple; protibiae slightly swollen to middle; mesotibiae with blunt preapical tubercle on mesal margin; metatibiae with small acute apical tubercle on mesal margin. Abdomen (Figs 5C, 22) with second ventrite depressed at medial fifth from middle to apex; third ventrite 0.60 wide, shallowly concave at medial third anterior to lamina, lamina 0.11 wide, apex broadly rounded, arising at point about two-thirds length of ventrite, strongly angled anteriorly at about 30°; fourth ventrite shallowly concave at medial third; fifth ventrite slightly depressed in basal third at middle; sixth ventrite (Fig. 5D) with posterior margin of setose area gradually narrowing to middle at about two-thirds lateral width. Aedeagus (Fig. 5A) 0.44 long; with parameres evenly rounded at apex; internal sac with single large acute spine at apex.

**Females:** Fifth tergite with setose area convex; fifth ventrite (Fig. 5F) with transverse margins of setose area parallel, irregular row of long setae present; ventrites all broadly convex at middle. Female genitalia (Fig. 5E) with median lobe elongate, membranous.

#### Collection notes.

This species was found in mixed leaf litter at a relatively cold and wet site within the Ponderosa pine zone of the Coast Ranges during the winter.

#### Geographical distribution.

(Map 1): This species is only known from western Tehama County in the Coast Ranges near the crest separating the eastern Sacramento River drainage from the western Eel River drainage.

#### Comparisons and diagnostic notes.

Placed as a member of the arcaps-group, and sharing with *Oropodes arcaps* and *Oropodes ishii* the anteriorly convex and medially placed lamina of the third ventrite. It is most similar to *Oropodes ishii* in sharing a preapical tubercle on the mesal margin of the mesotibiae and metatibiae with an apical spur in the males, and for the females the elongate membranous lobe of the genitalia without any spines. The males of these two species are easily separated by the nearly parallel margins of the setose area of the sixth ventrite in *Oropodes yollabolly*. The female characters are similar, and the species may be separated only by their distribution in the Coast Ranges, versus being in the northern Sierra Nevada for *Oropodes ishii*.

### The orbiceps-group.

Diagnostic features: Eyes larger, 45-65 facets. Males with basal spine on venter of profemora; second ventrite with posterior margin smooth, lacking teeth or lobes. Females with fifth tergite bearing two teeth at apex in the two species for which females are known; genitalia with membranous lobe nearly symmetrical.

**Map 2.**
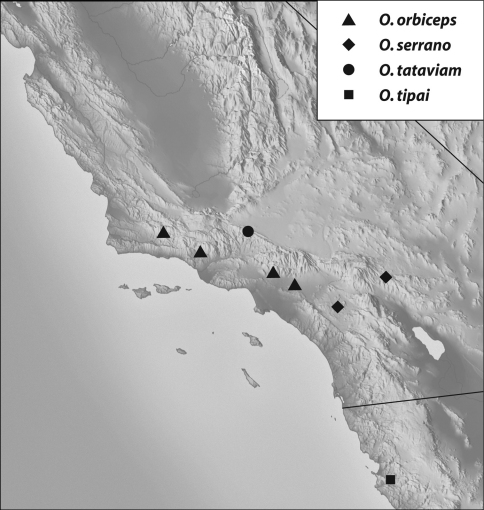
Records of the orbiceps-group: *Oropodes orbiceps, O. serrano, O. tataviam, O. tipai*.

Geographical distribution: The range of this group extends from the San Gabriel Mountains in southern California south to Baja California (Map 2).

Included species: *Oropodes orbiceps* Casey, *Oropodes serrano* sp. n., *Oropodes tataviam* sp. n., *Oropodes tipai* sp. n.

#### 5. Oropodes orbiceps

Casey, 1894

http://species-id.net/wiki/Oropodes_orbiceps

Fig. 6 24 Map 2

Oropodes orbiceps Casey, 1894: 453. Type locality: California: Los Angeles County. Holotype female: // S Cal/ Casey/ TYPE USNM 386111/ Oropodes orbiceps// (USNM). Raffray 1898: 246; 1904: 565; 1908: 81. Bowman 1934: 25. Grigarick & Schuster 1976: 99; 1980: pl. 29. Chandler 1997: 15. Not: Chandler 2003: 584.
Euplectus orbiceps : Fall 1901: 13.

##### Specimens examined.

25: CALIFORNIA: **Los Angeles Co.:** [county record], Holotype female (USNM); Pomona Mts., IV-30-1892, VI-4-1892, H.C. Fall Collection (1M, MCZC). Pasadena, A. Fenyes Collection (1M, 1F, UCDC). **Santa Barbara Co.:** LPNF [Los Padres National Forest], Sunset Valley, 34.7538°N, 119.9429°W, V-1-2004, M. Caterino, at light (5M, 7F, DSC, SBMN); UC Sedgwick Reserve, 34.7211°N, 120.0359°W, V-13-2005, M. Caterino, at light (1M, SBMN). **Ventura Co.:** Ojai, III-8-1892, H.C. Fall Collection (1M, 1F, MCZC); Ojai, III-8-1892, ex. Collection Rev. Jerome Schmitt (1890-1904)? St. Vincent Archabby (1M, 1F, FMNH). “Californie”, Museum Paris 1917 Coll. A. Raffray (3M, 1F, MNHN [undoubtedly sent to Raffray by Henry Fall]).

##### Description.

Length 1.76-1.92. Body orange-brown. Eyes in both sexes with about 60 facets. Antennomeres V and VII slightly larger than those adjacent, V-VIII obconical, IX smaller than X, antennae slender. Abdomen with carinae of first ventrite extending posteriorly from posteromedial angles of metacoxal cavities to apex.

**Figure 6.**
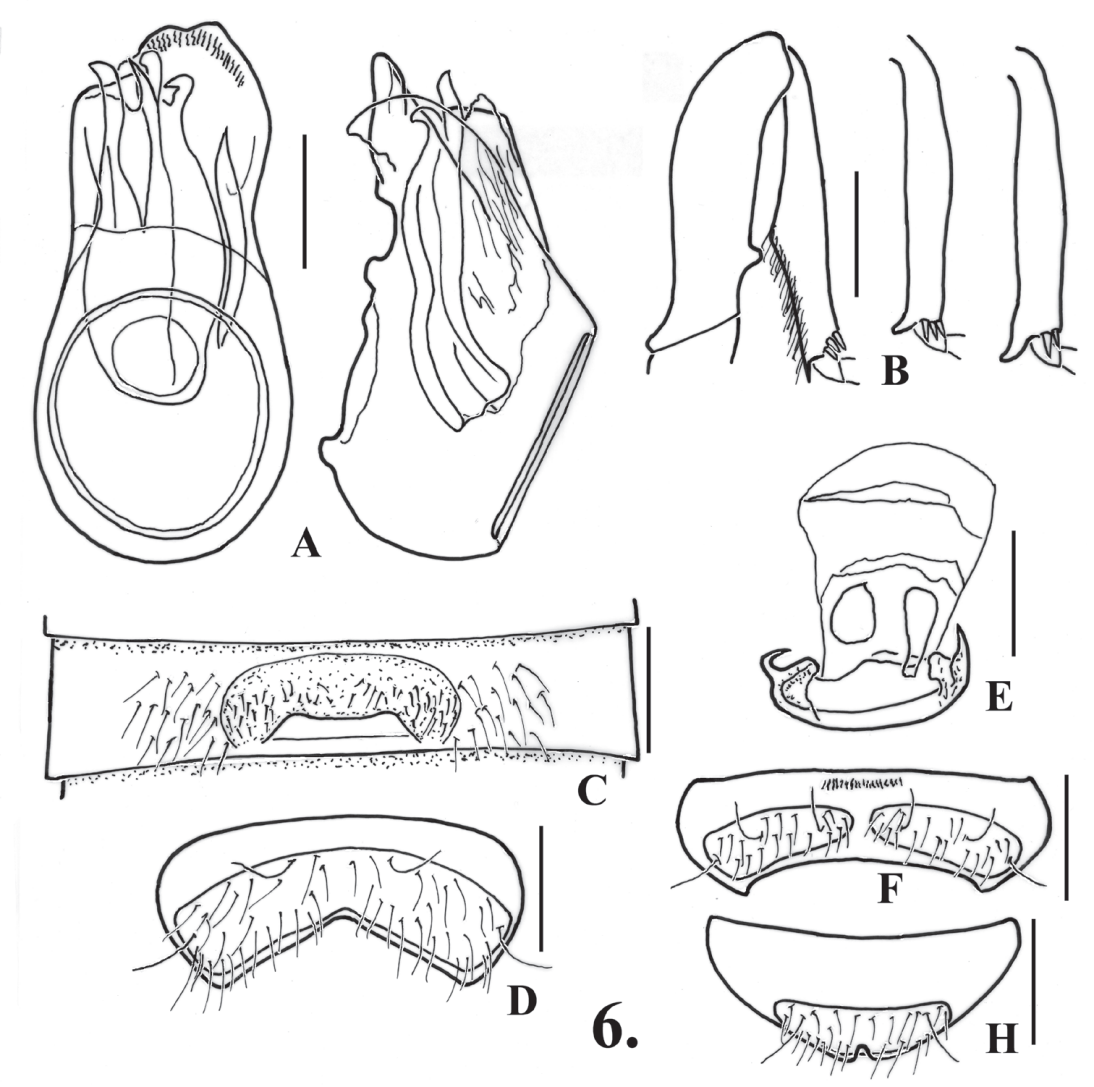
*Oropodes orbiceps* **A** Dorsal and right lateral view of male genitalia **B** Posterior view of right male profemur and protibia **C** Ventral view of male third ventrite **D** Ventral view of male sixth ventrite **E** Dorsal view of female genitalia **F** Dorsal view of female fifth ventrite. Scale line equals 0.1 mm.

**Males:** Metasternum with distinct median longitudinal sulcus. Legs (Fig. 6B): profemora with tubercle on mesal margin near base; protibiae with blunt angulation on mesal margin near middle; mesotibiae with straight apical spur on mesal margin; metatibiae with curved apical spur on mesal margin. Abdomen (Figs 6C, 24) with second ventrite gently concave in apical half to form semicircular glabrous impression in middle third, lacking teeth on posterior margin; third ventrite 0.47 wide, transverse recurved lamina 0.15 wide, lamina arising at ventrite apex, gradually curved at middle to about 25° angle from surface for anterior portion, transverse impression anterior to lamina densely setose, lacking well-defined margins; fourth-fifth ventrites flat in medial fourth, sixth ventrite (Fig. 6D) flat in medial fourth, anterior and posterior margins of setose area roughly parallel to middle where posterior margin is angulate. Aedeagus (Fig. 6A) 0.37 long; left paramere longer than right paramere, with apex sinuate, right paramere with apex subtrunctate; internal sac with two large spines, left spine forked near apex.

**Females:** Metasternum with faint median longitudinal sulcus. Fifth tergite with setose area convex, apex sharply emarginate, with two small separated apical teeth; fifth ventrite (Fig. 6F) with setose areas clearly separated by bar (Note: holotype female has the setose areas separated by a thin bar that is nearly interrupted apically. All other females have the bar distinct and complete). Genitalia (Fig. 6E) with large subrectangular median lobe widest near apex, with pair of elongate wide sclerites in basal portion that differ in size.

##### Collection notes.

Fall (1901) stated that he had collected this uncommon species in leaf litter at Pomona, Pasadena, and the Ojai Valley from March to June. All recently collected specimens were taken in May at light. The ‘Sunset Valley’ locality (Santa Barbara Co.), where a long series was taken, is an open valley oak (*Quercus lobata*) woodland.

##### Geographical distribution.

(Map 2): Found in the central and western portions of the Transverse Ranges ranging from the Santa Ynez Mountains near Santa Barbara to the San Gabriel Mountains near Pomona.

**Comparisons and diagnostic notes.** Placed in the orbiceps-group, whose members are characterized by a basal tooth on the profemora, the second ventrite lacks apical tubercles, and the two species for which females are known have two teeth at the apex of the fifth tergite. This species shares with *Oropodes tataviam* the medially angulate protibiae, and placement of the lamina at the posterior margin of the third ventrite in the males. The other two species placed in this group, *Oropodes serrano* and *Oropodes tipai* have the lamina positioned at about the two-thirds point of the ventrite length. *Oropodes orbiceps* has the male mesotibiae bearing an apical spur, and the female fifth ventrite has the setose area divided by a flat bar, while for *Oropodes tataviam* the male mesotibiae have widely separated preapical and medial tubercles, and the setose area of the female fifth ventrite is separated by an angular protrusion.

Records of *Oropodes orbiceps* from central and northern California are incorrect or probably so. The female specimen from Mt. Diablo in Contra Costa County (Grigarick and Schuster 1976) has not been located, but the identification is unlikely. Chandler (2003) also cites this species from Tehama County based on a female specimen, but examination of the female genitalia has revealed that it is a member of an undescribed species. A male specimen in the MNHN (Paris) is marked as “TYPE.” A note has been appended that it is not a type, since Casey’s description was based on the single specimen held in the USNM.

#### 6 Oropodes serrano

Chandler & Caterino sp. n.

urn:lsid:zoobank.org:act:F8F80139-8309-4D49-80C1-4BF7D86B8918

http://species-id.net/wiki/Oropodes_serrano

Fig. 7 Map 2

##### Specimens examined.

3: HOLOTYPE male, California, San Bernardino County, UC Burns [Piñon Ridge] Reserve, Railroad Canyon, 34.1405°N, 116.4541°W, IV-1-2008, M. Caterino & R. Leschen (SBMN, CBP0076208) PARATYPE, 1: eutopotypical (1M, DSC). Additional specimen: **Riverside County:** 1 mi S Bundy Canyon, nr. Menifee Valley, XI-28-1972, J.D. Pinto, UCRC ENT 00039640 (1M, UCRC).

##### Description.

(only males known): Length 1.68-1.84. Body light orange-brown. Eyes with about 45 facets. Antennomeres V and VII slightly larger than those adjacent, V-VIII obconical, IX smaller than X. Abdomen with carinae of first ventrite extending from posteromesal margins of metacoxal cavities to ventrite apex.

**Figure 7.**
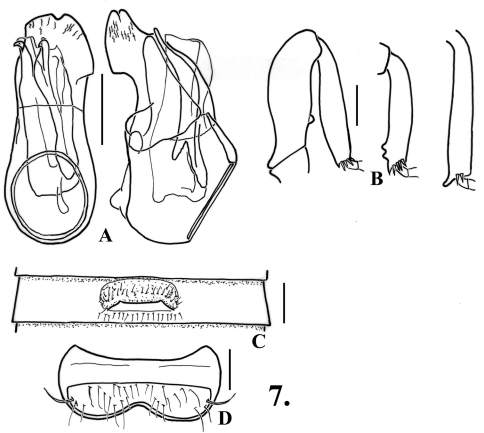
*Oropodes serrano* **A** Dorsal and right lateral view of male genitalia **B** Posterior view of right male protibia, mesotibia, and metatibia **C** Ventral view of male third ventrite **D** Ventral view of male sixth ventrite. Scale line equals 0.1 mm.

**Males:** Metasternum with broad shallow median longitudinal sulcus. Legs (Fig. 7B): profemora with blunt tooth near base on ventral margin; protibiae slightly swollen to point past middle, smooth on mesal margin; mesotibiae thickened and somewhat short, with two close rounded tubercles near apex on mesal maragin; metatibiae with curved apical spur on mesal margin. Abdomen (Fig. 7C) with second ventrite shallowly impressed in middle fourth to base; third ventrite 0.53 wide, with transverse impression in middle third anterior to recurved lamina, impression with irregular row of thickened setae, with row of setae posterior to lamina, lamina 0.15 wide, arising at point about two-thirds length of ventrite, apex nearly straight with lateral margins marked by small acute denticles, lamina angled at about 45°; fourth-fifth ventrites slightly impressed in middle third; sixth ventrite (Fig. 7D) flattened in middle third, setose area with posterior margin constricted toward middle. Aedeagus (Fig. 7A) 0.32 long; left paramere large, broadly rounded and with small indentation at apex; left paramere shorter and obscure; internal sac forming large tubular structure that is apically complex, with one large sinuate spine visible in lateral view.

##### Collection notes.

The two specimens from the U.C. Burns Reserve were taken in April by sifting clumps of grass roots alongside a small ephemeral stream.

##### Geographical distribution.

(Map 2): Found at the eastern end of the Transverse Ranges on the northeast side of the San Bernardino Mountains north of Yucca Valley, and ranging south to the Santa Ana Mountains near Elsinore.

##### Comparisons and diagnostic notes.

Placed in the orbiceps-group. Both *Oropodes serrano* and *Oropodes tipai* are most similar in the male protibiae being smooth on the mesal margins, and in placement of the lamina of the third ventrite at about the two-thirds point of the ventrite length. They may be separated by the lamina being angled at about 45° and the mesotibiae bearing two close preapical tubercles on the mesal margin in *Oropodes serrano*, while in *Oropodes tipai* the apical portion of the lamina is horizontal, and there is only one preapical tubercle on the mesotibiae. The females for both species are unknown. The specimen from Riverside County is larger (1.84 mm) than the other two (1.68-1.7 mm), with the lamina of the third ventrite appearing broader. However, the distinctive male genitalia and other male characters are similar.

##### Etymology.

The specific epithet, treated as a Latin singular noun in apposition, nominative case, is based on the tribal name of the Serrano Indians, who originally lived in the area where the holotype was taken.

#### 7 Oropodes tataviam

Chandler & Caterino sp. n.

urn:lsid:zoobank.org:act:ACFBF581-6685-45AA-8729-2BD2A487F9CC

http://species-id.net/wiki/Oropodes_tataviam

Fig. 8 Map 2

##### Specimens examined.

4: HOLOTYPE male, California, Los Angeles Co., Angeles National Forest, Hideaway Canyon, 34.6993°N, 118.5465°W, III-16/31-2007, Caterino & Chatzimanolis, FIT (SBMN; CBP0062414). PARATYPES, 3 females, same locality, each with a different date: IV-14/28-2007, V-14/28-2007, VI-13/23-2007 (DSC, SBMN).

##### Description.

Length 1.84-1.88. Body orange-brown. Eyes with about 65 facets. Antennomeres V and VII slightly larger than those adjacent, V-VIII obconical, IX smaller than X. Abdomen with carina at posteromesal margins of metacoxal cavities extending to first ventrite apex

**Figure 8.**
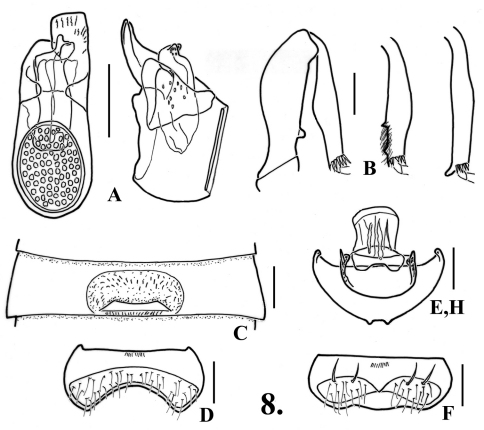
*Oropodes tataviam* **A** Dorsal and right lateral view of male genitalia **B** Posterior view of right male profemur and protibia, mesotibia, and metatibia **C** Ventral view of male third ventrite **D** Ventral view of male sixth ventrite **E** Dorsal view of female genitalia **F** Dorsal view of female fifth ventrite **H** Dorsal view female fifth tergite. Scale line equals 0.1 mm.

**Males**: Metasternum with shallow median longitudinal sulcus. Legs (Fig. 8B): profemora with blunt distinct tooth near base on mesal margin; protibiae with broad and rounded angulation on mesal margin at point past first half of length; mesotibiae with two small sharp tubercles in apical third on mesal margin, tubercles distant; metatibiae with curved apical spur on mesal margin. Abdomen (Fig. 8C) with ventrite 2 flattened in middle fourth; ventrite 3 0.55 wide, with broad transverse impression anterior to recurved lamina occupying middle third of ventrite, impression with short setae; lamina 0.17 wide, with apical margin slightly and broadly concave, arising at apex of ventrite, angled at about 35°; ventrites 4 and 5 barely flattened in middle third, ventrite 6 (Fig. 8D) flattened in middle fourth, setose area slightly narrowing to middle. Aedeagus (Fig. 8A) 0.29 long, left paramere longest, truncate at apex, left paramere subtruncate; internal sac with upturned lbe at apex, diaphragm with distinct spotted pattern of sclerotization.

**Females:** Tergite 5 with setose area of disc broadly convex; with pair of small teeth at apex (Fig. 8H), teeth not visible externally. Sternite 6 (Fig. 8F) with setose area divided by prominent projection from anterior portion, slightly overlapping posterior margin. Female genitalia (Fig. 8E) nearly symmetrical, with only slight size difference for two slender rods in the membranous median lobe.

##### Collection notes.

The four specimens were taken at a single site using a flight intercept trap from March to June, with a single specimen taken in each month. The trap site was located in a narrow ephemeral stream channel, surrounded by scrub oaks (*Quercus* spp.), gray or Digger pine (*Pinus sabiniana*), and mid-elevation chaparral.

##### Geographical distribution.

(Map 2): Found in the western portion of the San Gabriel Mountains at the northern edge bordering the Antelope Valley.

##### Comparisons and diagnostic notes.

Placed as a member of the orbiceps-group. It is closest to *Oropodes orbiceps* with the males sharing medially angulate protibiae, the metatibiae with a distinct apical spur, and the lamina of the third ventrite originates at the posterior margin, and the females have two apical teeth on the fifth tergite. They may be separated by the preapical and medial tubercles of the male mesotibiae of the males of *Oropodes tataviam* and for the females a protruding median tubercle divides the setose areas, versus an apical mesotibial spur for males of *Oropodes orbiceps*, and in females a flat bar or line divides the setose areas.

##### Etymology.

The specific epithet, treated as a Latin singular noun in apposition, nominative case, is based on the tribal name of the Tataviam Indians, who originally lived in the area where the specimens of this species were taken.

#### 8 Oropodes tipai

Chandler & Caterino sp. n.

urn:lsid:zoobank.org:act:F03C2086-7AF7-41B1-933E-9199DB966723

http://species-id.net/wiki/Oropodes_tipai

Fig. 9 Map 2

##### Specimen examined.

HOLOTYPE male, MEXICO, Baja California [Norte], El Uruapan, IV-2-1986, W. Clark, PE#5 (FMNH).

##### Description.

(only males known)**:** Length 2.16. Body brown. Eyes with about 65 facets. Antennomeres V and VII slightly larger than those adjacent, V-VIII obconical, IX narrower than X. Abdomen with carinae of first ventrite extending from posteromesal margins of metacoxal caviaties to ventrite apex.

**Figure 9.**
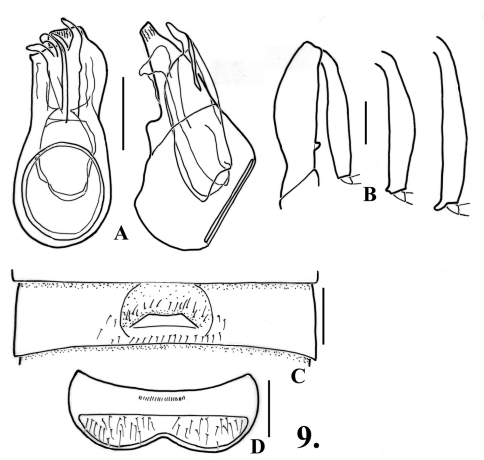
*Oropodes tipai* **A** Dorsal and right lateral view of male genitalia **B** Posterior view of right male profemur and protibia, mesotibia, and metatibia **C** Ventral view of male third ventrite **D** Ventral view of male sixth ventrite. Scale line equals 0.1 mm.

**Males**: Metasternum with shallow median longitudinal sulcus. Legs (Fig. 9B): profemora with blunt spine on ventral margin near base, protibiae slightly swollen to middle, inner margin smooth; mesotibiae with small preapical tubercle; metatibiae with curved apical spur on mesal margin. Abdomen (Fig. 9C) with second ventrite impressed in middle third to form semicircular impression extending anteriorly from ventrite apex; third ventrite 0.51 wide, with transverse impression in median third of width, lateral margins of impression carinate, impression with transverse zone of thin setae, lamina arising at about two-thirds point of ventrite length and projecting anteriorly over impression, lamina 0.12 wide, apex broadly and shallowly emarginate, lamina slightly curved at base, apical portion straight and nearly horizontal; fourth-fifth ventrites lightly impressed in middle third; sixth ventrite (Fig. 9D) with setose area constricted at middle to about half maximum width. Aedeagus (Fig. 9A) 0.32 long, with left paramere longest, apices of both parameres truncate; internal sac with several laterally curved spines.

**Females**: unknown.

##### Collection notes.

Taken in March.

##### Geographical distribution.

(Map 2): The single record is from the western side of the Sierra de San Pedro Martír in Mexico, about 140 km south of the United States border.

##### Comments.

Placed in the orbiceps-group. Both *Oropodes tipai* and *Oropodes serrano* are most similar in the male protibiae being smooth on the mesal margins, and in placement of the lamina of the third ventrite at about the two-thirds point of the ventrite length. They may be separated by the lamina being nearly horizontal in the apical portion and the mesotibiae bearing only one preapical tubercle on the mesal margin in *Oropodes tipai*, while in *Oropodes serrano* the lamina of the third ventrite is angled at about 45°, and there are two close preapical tubercles on the mesotibiae. The females for both species are unknown.

##### Etymology.

The specific epithet, treated as a Latin singular noun in apposition, nominative case, is based on the tribal name of the Tipai Indians who originally lived in northern Baja California, the area where the holotype of this species was taken.

### The raffrayi-group.

Diagnostic features: Eyes larger, 50-70 facets. Males with basal spine on venter of profemora; second ventrite bearing two teeth or lobes spaced about as wide as lamina of third ventrite. Females lacking teeth on apical margin of fifth tergite; genitalia with membranous lobe nearly symmetrical to asymmetrical.

Geographical distribution: The range of this group extends from northern California to the San Gabriel Mountains of southern California (Maps 1 and 2).

Included species: Ten species, forming two clusters based on position of the male lamina of the third ventrite. Lamina on posterior margin: *Oropodes bellorum* sp. n., *Oropodes esselen* sp. n., *Oropodes hardyi* sp. n., *Oropodes nuclere* Grigarick & Schuster, *Oropodes raffrayi* (Brendel), *Oropodes rumseyensis* Grigarick & Schuster, and *Oropodes tongva* sp. n. Lamina median: *Oropodes aalbui* sp. n., *Oropodes casson* sp. n., and *Oropodes chumash* sp. n.

#### 8 Oropodes aalbui

Chandler & Caterino sp. n.

urn:lsid:zoobank.org:act:D4D8A36B-5CFB-448A-927C-37AEA5504938

http://species-id.net/wiki/Oropodes_aalbui

Fig. 10 Map 3

##### Specimen examined.

HOLOTYPE male, California, San Bernardino County, Mexican Mine [34.9491°N, 115.5103°W], 4200’, XII-31-1978/VI-16-1979, R.L. Aalbu (CSCA).

##### Description.

(only holotype male known): Length 2.50. Body orange-brown. Eyes with about 50 facets. Antennomeres V and VII slightly larger than those adjacent, V-VIII obconical, IX smaller than X. Abdomen with carinae on first ventrite extending from posteromesal margins of metacoxal cavities to ventrite apex.

**Figure 10.**
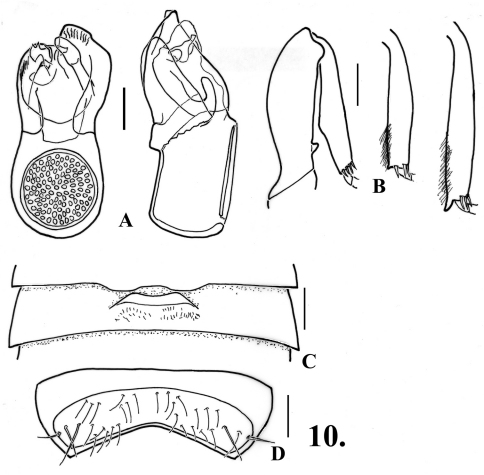
*Oropodes aalbui* **A** Dorsal and right lateral view of male genitalia **B** Posterior view of right male profemur and protibia, mesotibia, and metatibia **C** Ventral view of male third ventrite **D** Ventral view of male sixth ventrite. Scale line equals 0.1 mm.

**Males:** Legs (Fig. 10B): profemora with distinct angled tooth near base on ventral margin; protibiae with blunt angular expansion widest at beginning of apical third of length; mesotibiae with small acute tubercle at apex on mesal margin; metatibiae with small curved spur at apex on mesal margin. Metasternum with sharply defined median longitudinal sulcus, lateral margins of sulcus carinate. Abdomen (Fig. 10C) with second ventrite shallowly and semicircularly concave in middle fourth, impression extending anteriorly from lateral margins of two broad, shallow teeth on apical margin, center of teeth about 0.15 apart; third ventrite 0.60 wide, with recurved lamina at middle, lamina 0.20 wide, apex broadly and shallowly concave, angled at about 25°, dense short setae in transverse zone posterior to lamina; fourth and fifth ventrites shallowly impressed in middle fourth; sixth ventrite (Fig. 10D) with anterior/posterior margins of setose area nearly parallel. Aedeagus (Fig. 10A) 0.49 mm long; left paramere longest, broadly and irregularly rounded at apex, right paramere more narrowly rounded, internal sac with heavily sclerotized armature in apical portion.

**Females:** unknown.

##### Collection notes.

Taken using a pitfall trap with antifreeze preservative over a six-month period deep in a mine in southern California. The area surrounding the mine is high-elevation desert scrub (*pers. comm.* Rolf L. Aalbu).

##### Geographical distribution.

**Map 3.**
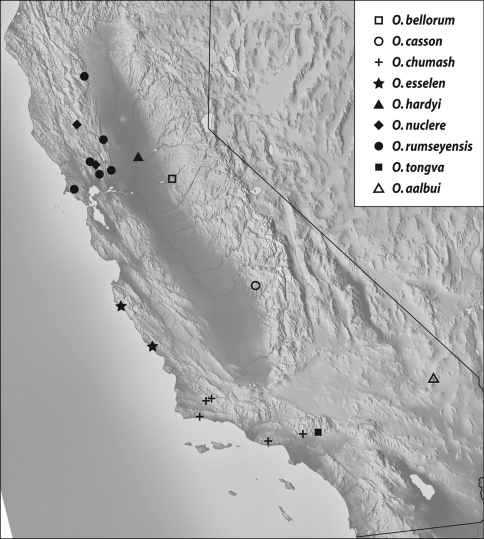
Records of the raffrayi-group: *Oropodes aalbui*, *Oropodes bellorum*, *Oropodes casson*, *Oropodes chumash*, *Oropodes esselen*, *Oropodes hardyi*, *Oropodes nuclere*, *Oropodes rumseyensis*, *Oropodes tongva*.

(Map 3): The Providence Mountains Recreation Area is isolated in the eastern Mojave Desert.

##### Comparisons and diagnostic notes.

Placed as a member of the raffrayi-group, and most similar to *Oropodes casson* and *Oropodes chumash* in sharing the median position of the lamina of the third ventrite and the blunt or rounded apical projections of the second ventrite. The armature of all the tibiae is close to that of *Oropodes casson*: protibiae medially angulate, and relatively short apical spurs of the meso- and metatibiae. The lamina of the third ventrite is more reflexed (at about 25°) than that of *Oropodes casson* (at about 40°), the projections of the second ventrite are broadly rounded lobes versus more prominent in *Oropodes casson*, and the armature of the internal sac is not as spinose.

##### Etymology.

The specific epithet is a singular Latinized noun in the genitive case, based on the surname of Rolf L. Aalbu of Sacramento, coleopterist and computer programming consultant, who collected the holotype in an area where pselaphine collectors would never think to venture.

#### 10. Oropodes bellorum

Chandler & Caterino sp. n.

urn:lsid:zoobank.org:act:C7F28649-A585-434B-93FA-669C50AEF6A7

http://species-id.net/wiki/Oropodes_bellorum

Fig. 11 Map 3

##### Specimens examined.

2: HOLOTYPE male, California, Calaveras County, 3 mi S Mokelumne Hill, II-3/V-7-1981, S. Kuba & R. Aalbu, antifreeze pit trap (CSCA). PARATYPE male, eutopotypical (DSC).

##### Description.

(only males known): Length 2.28. Body orange-brown. Eyes with about 70 facets. Antennomeres V and VII slightly larger than those adjacent, V-VIII obconical, IX nearly as large as X. Abdomen with carinae on first ventrite extending from posteromedial angles of metacoxal cavities to ventrite apex.

**Figure 11.**
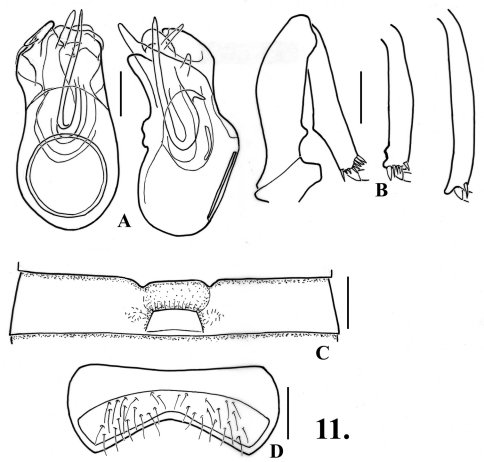
*Oropodes bellorum* **A** Dorsal and right lateral view of male genitalia **B** Posterior view of right male profemur and protibia, mesotibia, and metatibia **C** Ventral view of male third ventrite **D** Ventral view of male sixth ventrite. Scale line equals 0.1 mm.

**Males**: Metasternum with median longitudinal sulcus distinct across most of disc. Legs (Fig. 11B): protrochanters with protruding broad ventral lamina; profemora with blunt tooth near base on mesal margin; protibiae with mesal angulation at point about two-thirds of length; mesotibiae with two small blunt close preapical tubercles on mesal margin; metatrochanters with posterior margin angulate, metatibiae with apical spur on mesal margin. Abdomen (Fig. 11C) with second ventrite semicircularly impressed between apical teeth to middle of ventrite, impression about one-fourth of tergite width, apical teeth sharp, 0.11 apart; third ventrite 0.62 wide, slightly impressed anterior to recurved lamina, lamina 0.11 long, with long setae originating beneath, short setae clustered laterally within impression, lamina abruptly curved at middle with apical portion horizontal, apical margin straight, lamina originating at posterior margin; fourth-fifth ventrites flattened at medial third; sixth ventrite (Fig. 11D) convex, setose area slightly narrowing to middle where half lateral length. Aedeagus (Fig. 11A) 0.46 long, with parameres about same length; complex armature of internal sac obscuring details, but with at least one long recurved spine.

**Females**: unknown.

##### Collection notes.

Taken in late winter/early spring from pitfall traps in a dry scrub forest area of the Sierra Nevada foothills.

##### Geographical distribution.

(Map 3): The single record is from the west side of the middle portion of the Sierra Nevada.

##### Comparisons and diagnostic notes.

Placed as a member of the raffrayi-group, and shares with six other species the origin of the abdominal lamina at the posterior margin of the third ventrite. The lamina in the other species has a concave anterior margin and they are comparatively thin, while in *Oropodes bellorum* the lamina is trapezoidal with the anterior margin straight, and is comparatively robust at about half as long as wide. This males of this species share with *Oropodes hardyi* the lamina being horizontal in the apical portion, the modified ventral margin of the protrochanters, the angulate mesal margin of the protibiae, and two close preapical teeth on the mesotibiae. These two species may be separated by the trapezoidal lamina, broad truncate tubercle on the protrochanters, and the angulate posterior margin of the metatrochanters of *Oropodes bellorum*, while in *Oropodes hardyi* the lamina is narrow with the anterior margin concave, the protrochanters have a small apical lobe, and the posterior margin of the metatrochanters are smoothly convex.

##### Etymology.

The specific epithet is a Latinized genitive plural noun, based on the surname of Ross T. and Joyce R. Bell, intended to honor them for their contributions to the study of Coleoptera.

#### 11. Oropodes casson

Chandler & Caterino sp. n.

urn:lsid:zoobank.org:act:36E1D0D6-0799-4C88-9012-3A174EC037C6

http://species-id.net/wiki/Oropodes_casson

Fig. 12 Map 3

##### Specimen examined.

HOLOTYPE male: California, Sequoia National Park, Ash Mt. Road, IV-30-1955, H.R. Moffitt (UCDC).

##### Description.

Length about 1.90. Body light orange-brown. Eyes with about 65 facets. Antennomeres V and VII slightly larger than those adjacent, V-VIII obconical, IX nearly as large as X. Abdomen with carinae of first ventrite extending from posteromesal margin of metacoxal cavities posteriorly to ventrite apex.

**Figure 12.**
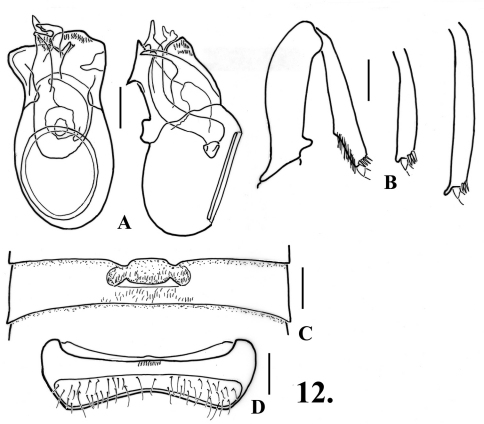
*Oropodes casson* **A** Dorsal and right lateral view of male genitalia **B** Posterior view of right male profemur and protibia, mesotibia, and metatibia **C** Ventral view of male third ventrite **D** Ventral view of male sixth ventrite. Scale line equals 0.1 mm.

**Males:** Metasternum with median longitudinal sulcus. Legs (Fig. 21): profemora with large tubercle near base on mesal margin; protibiae angulate on mesal margin at about two-thirds length from base; mesotibiae with short apical spur; metatibiae with apical spur. Abdomen (Fig. 12C) with disc of second ventrite semicircularly impressed to two blunt teeth on apical margin, teeth 0.12 apart; third ventrite 0.58 wide, with disc transversely impressed anterior to narrow lamina, impression with irregular row of short dense setae, lamina 0.16 wide, originating near middle of ventrite, deeply emarginate on anterior margin, lamina raised at about 40°; fourth-sixth ventrites flattened in middle third; setose area of sixth ventrite (Fig. 12D) slightly narrowing toward medially, still well-separated at middle. Aedeagus (Fig. 12A) 0.48 long; with left paramere angularly rounded, longer than subtruncate right paramere, with two curving spines that are apically divided and complex.

**Females:** unknown.

##### Collection notes.

Taken in late April from a dry-scrub forest area.

##### Geographical distribution.

(Map 3): The single record is from the west side of the southern portion of the Sierra Nevada.

**Comparisons and diagnostic notes**. Placed as a member of the raffrayi-group, and most similar to *Oropodes aalbui* and *Oropodes chumash* in sharing the median position of the lamina of the third ventrite and the blunt or rounded apical projections of the second ventrite. The armature of all the tibiae is close to that of *Oropodes aalbui*: protibiae medially angulate, and relatively short apical spurs of the meso- and metatibiae. The lamina of the third ventrite is more upright (at about 40°) than that of *Oropodes aalbui* (at about 25°), the projections of the second ventrite are more prominent versus the broadly rounded lobes found in *Oropodes aalbui*, and the large rods of the internal sac are more spinose in the apical portion. This specimen was originally placed as *Oropodes nuclere* by Grigarick & Schuster (1976).

##### Etymology.

The specific epithet, treated as a Latin singular noun in apposition, nominative case, is based on the tribal name of the Casson group of Yokut Indians, who originally lived in the area where the holotype of this species was taken.

#### 12. Oropodes chumash

Chandler & Caterino sp. n.

urn:lsid:zoobank.org:act:445E8A8E-7F4C-49FB-82AA-13991B6B8EC4

http://species-id.net/wiki/Oropodes_chumash

Fig. 1 13 25 Map 3

##### Specimens examined.

84: HOLOTYPE male:California, Santa Barbara Co., Arroyo Hondo Preserve, 25 mi W Santa Barbara, 34.4839°N, 120.1424°W, IV-16/28-2003, M. Caterino, FIT (SBMN; CBP0008565). PARATYPES: **Santa Barbara Co.:** Arroyo Hondo Preserve, 25 mi W Santa Barbara, IV-24/29-2002 (1M, 7F), IV-29/V-6-2002, FIT(1M, 5F), V-13/20-2002 (1M, 3F), V-20/27-2002 (1M), VI-3/12-2002 (1M), XI-11/27-2002 (1M), III-19/IV-2-2003 (1M), IV-2/16-2003 (1M), IV-16/28-2003 (1M, 1F), V-7/21-2003 (1F), V-21/28-2003 (1M), VI-11/VII-2-2003 (1F), M. Caterino, FIT; same data except, IV-29/V-6-2002, carrion pitfall (1) (topotypical paratypes in SBMN, LACM, CASC, UCDC, EMUS). UC Sedgwick Reserve, V-2/14-2005 (5M, 1F), V-14/29-2005 (2M), VI-12/26-2005 (2M), M. Caterino, FIT; same data except, V-13-2005 (5M, 3F), at light (all SBMN); Los Padres National Forest, Sunset Valley, V-1-2004, M. Caterino, at light (1F, SBMN). **Los Angeles Co.:** Santa Monica Mountains NRA, Rocky Oaks, IV-19-2009, M.S. Caterino & K.J. Hopp, at light (13M, 21F, DSC and SBMN). Pasadena, A. Fenyes Collection (1F, UCDC).

##### Description.

Length 1.84-2.08. Body orange-brown. Eyes with 50-60 facets, female eyes appearing slightly smaller than those of males. Antennomeres V and VII slightly larger than those adjacent, V-VIII obconical, IX smaller than X. Abdomen with carinae of first ventrite extending from posteromesal margin of metacoxal cavities posteriorly to ventrite apex.

**Figure 13.**
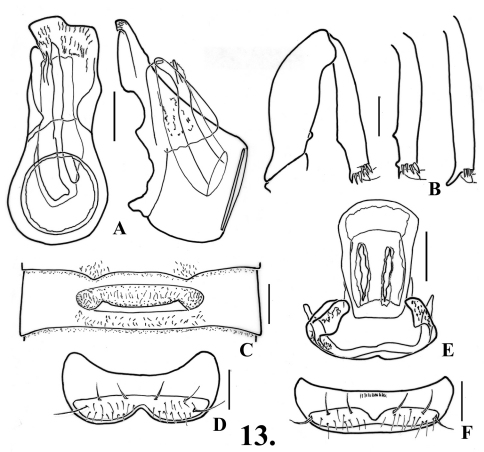
*Oropodes chumash* **A** Dorsal and right lateral view of male genitalia **B** Posterior view of right male profemur and protibia, mesotibia, and metatibia **C** Ventral view of male third ventrite **D** Ventral view of male sixth ventrite **E** Dorsal view of female genitalia **F** Dorsal view of female fifth ventrite. Scale line equals 0.1 mm.

**Males**: Metasternum with broad median longitudinal sulcus to near apex. Legs (Fig. 13B) profemora with low tubercle on mesal margin near base; protibiae with slight preapical angulation on mesal margin; mesotibiae with widely separated sharp preapical and blunt apical tubercles on mesal margin; metatibiae with long apical spur on mesal margin. Abdomen (Figs 13C, 25) with second ventrite bearing two broad teeth at apex, teeth 0.2 apart at centers, semicircular area anterior to teeth on ventrite 1 deeply impressed and glabrous; third ventrite 0.62 wide, broadly impressed in middle third anterior to wide transverse lamina near center of ventrite, lamina 0.24 wide, apex of lamina broadly concave, lamina curves at base to about 25°, transversely oval impression anterior to lamina with row of thickened setae across middle, lateral margins of impressed area carinate, area posterior to lamina with dense short setae; fourth ventrite shallowly concave at middle third; fifth ventrite obscurely flattened at middle third; sixth ventrite (Fig. 13D) briefly flattened at middle, setose area sharply constricted at middle. Aedeagus (Fig. 13A) 0.36 long; right paramere longest, broad apex sinuate, left paramere much smaller, bluntly rounded at apex; internal sac with single elongate blunt spine, spine sinuate in lateral view.

**Females:** Metasternum with thin and faint median longitudinal sulcus through most of length. Fifth tergite with setose area convex. Sixth ventrite (Fig. 13F) with setose area usually divided by projection from anterior margin, closure complete to slightly interrupted, projection protruding posteriorly. Genitalia (Fig. 13E) symmetrical or nearly so, with two irregular narrow sclerotized rods in large membranous subrectangular median lobe.

##### Collection notes.

Most of the specimens were taken in flight intercept traps set in coastal semiriparian woodland. Trapping localities were dominated by bay (*Umbellularia*) and live oak (*Quercus agrifolia*), with a nearby gallery forest of sycamore (*Platanus*), alder (*Alnus*), and willow (*Salix*). A few specimens were collected in drier, upland oak woodland and chaparral (xeric shrubland dominated by *Ceanothus*, *Rhus*, *Rhamnus*, and other *Quercus* spp.) and at ultraviolet light. Adults were active from March to July, with one record from November.

##### Geographical distribution.

(Map 3): Found in the western and central portions of the Transverse Ranges from the San Rafael Mountains north of Santa Barbara to the San Gabriel Mountain near Pasadena.

##### Comparisons and diagnostic notes.

Placed as a member of the raffrayi-group, and most similar to *Oropodes aalbui* and *Oropodes casson* in sharing the median position of the lamina of the third ventrite and the blunt or rounded apical projections of the second ventrite. The armature of all the tibiae is different from both of these species: the protibiae are not obviously medially angulate, the mesotibiae have widely separated apical and preapical tubercles, and the metatibiae have a long apical spur. The lamina of the third ventrite is similarly reflexed (at about 25°) as that of *Oropodes aalbui* but appearing much wider. The male sixth ventrite is constricted medially versus even in width for the other two species, and the armature of the internal sac lacks any apical spines. Of the three species discussed here, *Oropodes chumash* is the only one with associated females.

##### Etymology.

The specific epithet, treated as a Latin singular noun in apposition, nominative case, is based on the tribal name of the Chumash Indians, who originally lived in the area where the specimens of this species were taken.

#### 13. Oropodes esselen

Chandler & Caterino sp. n.

urn:lsid:zoobank.org:act:A41E5AE0-EB95-4250-8F42-1DF9A625CCD0

http://species-id.net/wiki/Oropodes_esselen

Fig. 14 Map 3

##### Specimens examined.

6: HOLOTYPE male: California, Monterey Co., UC Big Creek Reserve, Canogas Falls, 36.0616°N, 121.5545°W, III-27/IV-2-2004, M. Caterino, FIT (SBMN; CBP0018645). PARATYPES: eutopotypical (1M, 3F, DSC, SBMN). San Luis Obispo Co.: UC Rancho Marino Res., 35.5249N, 121.0719W, II-26-2009, M.S. Caterino, *Salix* litter (1M, SBMN).

##### Description.

Length 1.68-2.12. Body orange-brown. Eyes with 65-70 facets. Antennomeres V and VII slightly larger than those adjacent, IX smaller than X. Abdomen with first ventrite bearing carinae that extend from posteromedial angles of metacoxal cavities to ventrite apex.

**Figure 14.**
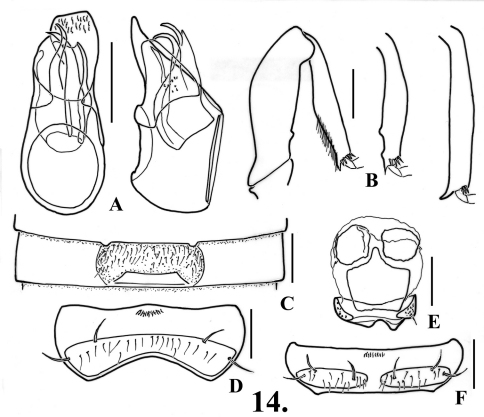
*Oropodes esselen* **A** Dorsal and right lateral view of male genitalia **B** Posterior view of right male profemur and protibia, mesotibia, and metatibia **C** Ventral view of male third ventrite **D** Ventral view of male sixth ventrite **E** Dorsal view of female genitalia **F** Dorsal view of female fifth ventrite. Scale line equals 0.1 mm.

**Figure 15.**
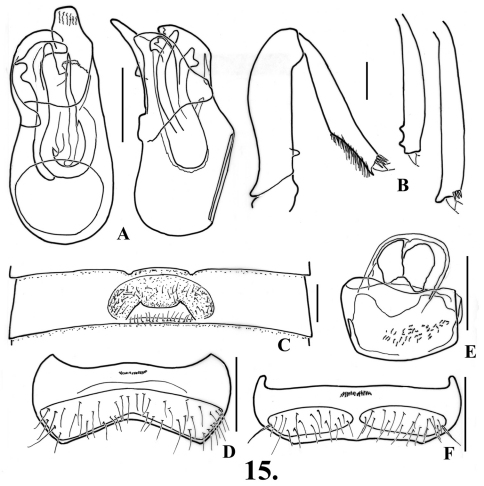
*Oropodes hardyi* **A** Dorsal and right lateral view of male genitalia **B** Posterior view of right male profemur and protibia, mesotibia, and metatibia **C** Ventral view of male third ventrite **D** Ventral view of male sixth ventrite **E** Dorsal view of female genitalia **F** Dorsal view of female fifth ventrite. Scale line equals 0.1 mm.

**Figure 16.**
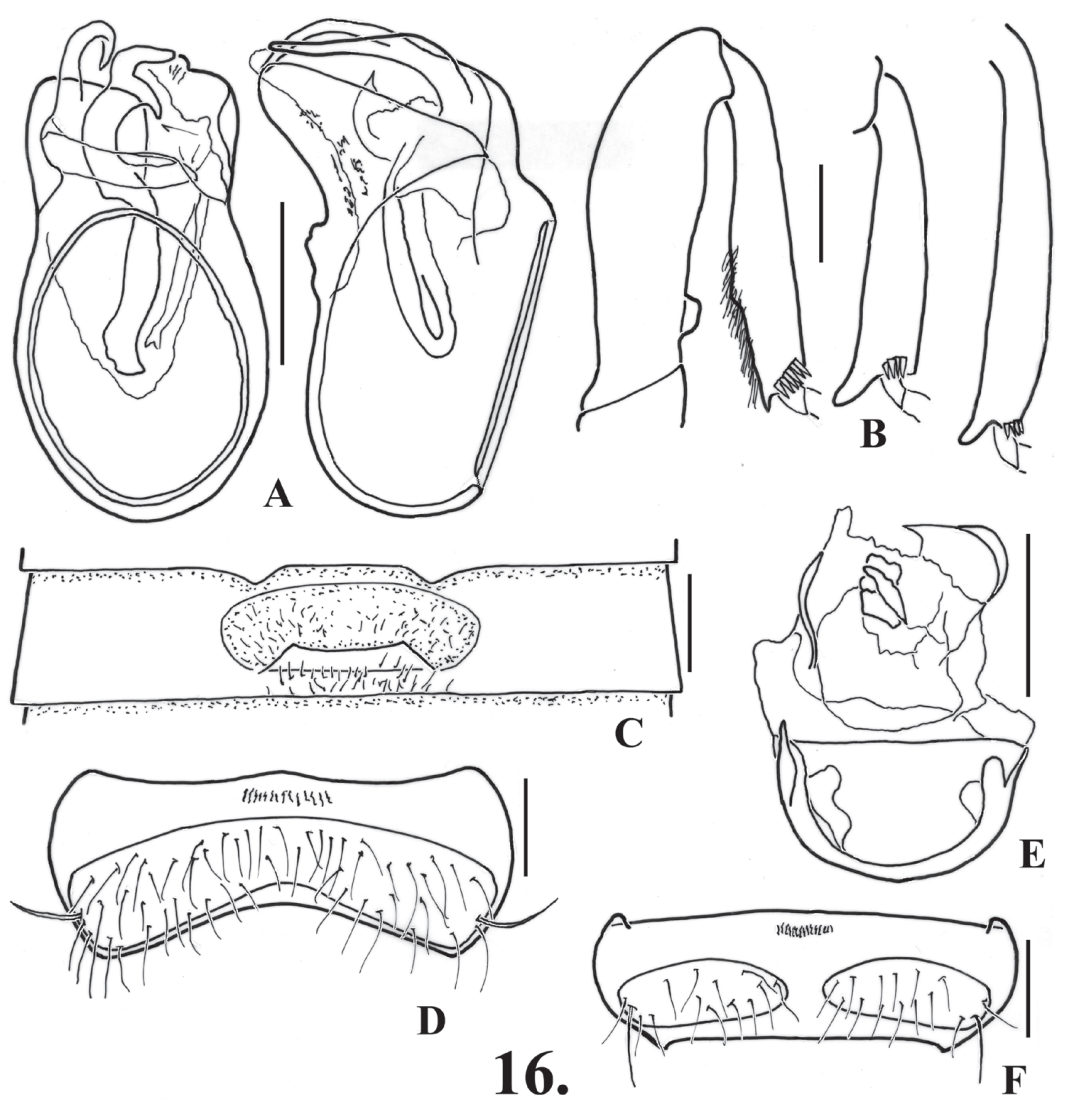
*Oropodes nuclere* **A** Dorsal and right lateral view of male genitalia **B** Posterior view of right male profemur and protibia, mesotibia, and metatibia **C** Ventral view of male third ventrite **D** Ventral view of male sixth ventrite **E** Dorsal view of female genitalia **F** Dorsal view of female fifth ventrite. Scale line equals 0.1 mm.

**Males**: Metasternum convex. Legs (Fig. 14B): profemora with small ventral tooth near base; protibiae swelling slightly to middle; mesotibia with widely-separated blunt preapical and apical tubercles on mesal margin; metatibiae with curved apical spur on mesal margin. Abdomen (Fig. 15C) with second ventrite gently concave in area anterior to two small teeth at apex, teeth 0.18 apart at centers; third ventrite 0.57 wide, with broad recurved lamina originating at apex of ventrite, lamina 0.14 wide, apex slightly concave, angled at about 30°, transverse impressed area anterior to lamina with scattered thickened setae, defined laterally by carinae, lacking setae posterior to lamina; fourth ventrite gently concave in medial third; fifth ventrite slightly convex/flattened in medial fourth; sixth ventrite (Fig. 15D) gently convex at middle, transverse margins of setose area slightly narrowing toward middle. Aedeagus (Fig. 16A) 0.27 long; with left paramere prominent and laminate, right paramere shorter and broadly rounded; with two elongate spines in internal sac curved to right apically.

**Females**: Fifth ventrite (Fig. 15F) with setose area broadly divided by medial bar. Genitalia (Fig. 15E) symmetrical, median lobe with broad medial plate apically pointed.

##### Collection notes.

A small series was taken using a flight intercept trap from late March to early April. The type locality was near a small ephemeral stream in a chaparral area, with *Ceanothus*, *Heteromeles*, *Arctostaphylos*, and small *Quercus*. A single male was taken by sifting willow litter in February.

##### Geographical distribution.

(Map 3): Taken from foothills of the Coast Ranges along the coast in Monterey and San Luis Obispo Counties.

##### Comparisons and diagnostic notes.

A member of the raffrayi-group, and sharing with six other species the abdominal lamina arising at the posterior margin of the third ventrite. This species is distinct in this group by the males having a small basal profemoral tooth, unmodified protibiae, and mesotibiae with the preapical tubercles widely separated, while for females the genitalia is symmetrical, and the fifth ventrite has the setose area widely divided by a bar. This is the only species where the male metasternum is convex, rather than with a median longitudinal sulcus.

##### Etymology.

The specific epithet, treated as a Latin singular noun in apposition, nominative case, is based on the tribal name of the Esselen Indians, who originally lived in the area where the specimens of this species were taken.

#### 14. Oropodes hardyi

Chandler & Caterino sp. n.

urn:lsid:zoobank.org:act:03961B32-318F-48FB-8D1E-69DE98D53A67

http://species-id.net/wiki/Oropodes_hardyi

Fig. 15 Map 3

##### Specimens examined.

5: HOLOTYPE male: California, Sacramento County: Sacramento, Hillsdale, IV-14-1980, A.R. Hardy, B-lite (CSCA). PARATYPES: 1 male, Sacramento, Hillsdale, IV-15-1980, A.R. Hardy (CSCA); 2 males, 1 female, Sacramento, Hillsdale area, V-17/19-1980, A. Hardy (CSCA, DSC).

##### Description.

Length 2.36-2.40. Body light orange-brown. Eyes with around 60-65 facets. Antennomeres V and VII slightly larger than those adjacent, V-VIII obconical, IX nearly as large as X. Abdomen with first ventrite either lacking carinae that extend from posteromedial angles of metacoxal cavities to ventrite apex, or carinae faint.

**Males:** Metasternum with median longitudinal sulcus. Legs (Fig. 15B): protrochanters with small lobe on ventral margin; profemora with prominent tooth near base on mesal margin; protibiae thickening from base to blunt angulation at about apical two-thirds; mesotibiae with two close preapical angulations on mesal margin; metatibiae with apical spur on mesal margin. Abdomen (Fig. 15C) with second ventrite shallowly impressed in middle fourth from middle of ventrite to pair of teeth on apical margin, teeth 0.35 apart at centers; third ventrite 0.71 wide, with transversely oval impression with scattered short setae anterior to transverse median lamina, lamina 0.18 wide, broadly emarginate at apex, in lateral view evenly curved at base with apical portion nearly horizontal, lamina arising at posterior margin of ventrite; fourth-sixth ventrites flattened in medial third; sixth ventrite (Fig. 15D) with anterior/posterior margins of setose area slightly narrowing to middle. Aedeagus (Fig. 15A) 0.35 long, with left paramere protruding and laminate, right paramere barely visible in dorsal view and broadly rounded at apex, internal sac with at least two elongate spines bearing bluntly rounded tips, right one with lateral tubercles before apex.

**Females:** Fifth tergite with setose area convex, with small impression at apex. Fifth ventrite (Fig. 15F) with setose area narrowly divided by median glabrous bar. Genitalia (Fig. 15E) possibly symmetric, perhaps skewed during extraction, median lobe with large longitudinally divided sclerite.

##### Collection notes.

All specimens were taken at blacklight in a backyard within a residential area without any nearby patches of native vegetation nearby (*pers. comm*. Alan Hardy). The specimens were collected in April and May.

##### Geographical distribution.

(Map 3): This species was found in urban Sacramento, in the low foothills of the Sierra Nevada near the valley floor of the Sacramento Valley.

##### Comparisons and diagnostic notes.

Placed as a member of the raffrayi-group, and sharing with six species the origin of the abdominal lamina at the posterior margin of the third ventrite. This species is most similar to *Oropodes bellorum* from Calaveras County in sharing the apical portion of the lamina being horizontal, the modified ventral margin of the protrochanters, the angulate mesal margin of the protibiae, and two close preapical teeth on the mesotibiae. These two species may be separated by the thin lamina with the concave anterior margin, the small apical lobe on the protrochanters, and smoothly convex posterior margin of the metatrochanters for *Oropodes hardyi*, while *Oropodes bellorum* has a robust trapezoidal lamina, a broad, truncate tubercle on the protrochanters, and the posterior margin of the metatrochanters is angulate.

##### Etymology.

The specific epithet is a Latinized singular noun in the genitive case, based on the surname of Alan R. Hardy, retired coleopterist from the California Department of Agriculture, Sacramento, who collected the type series of this species.

#### 15. Oropodes nuclere

Grigarick & Schuster, 1976

http://species-id.net/wiki/Oropodes_nuclere

Fig. 16 Map 3

Oropodes nuclere Grigarick & Schuster, 1976: 105; Chandler 1997: 15. Type locality: California, Lake County, Lucerne. Holotype male (UCDC).

##### Specimens examined.

3: CALIFORNIA: **Lake County:** Lucerne, #99, VI-1-1961, R.O. Schuster (UCDC, 2 paratype females). **Napa County:** Rutherford, V-26-1966, W.C. Gagne (DSC).

##### Description.

Length 1.90-2.00 mm. Body light orange-brown. Antennomeres V and VII slightly larger than those adjacent. Eyes with about 70 facets in both sexes. Abdomen with first ventrite bearing carinae that extend posteriorly from posteromedial angles of metacoxal cavities to apex.

**Males**: Metasternum with median longidudinal sulcus. Legs (Fig. 16B): profemora with broad mesal tooth near base; protibiae lacking modifications; meso- and metatibiae with prominent apical spur on mesal margin, spur of metatibiae smaller than that of mesotibiae. Abdomen (Fig. 16C, interpreted from specimen on slide) with shallow impression at middle between two widely spaced teeth at second ventrite apex, teeth 0.18 apart at centers; third ventrite 0.71 wide, impressed in middle third, lamina 0.18 wide, broadly emarginate at apex, angled at about 30°, close to posterior margin, oval transverse impression anterior to lamina with short setae; fourth ventrite slightly impressed in middle fourth; fifth-sixth ventrites lightly impressed in medial fourth; sixth ventrite (Fig. 16D) with margins of setose area slightly narrowed at middle. Aedeagus (Fig. 16A) 0.32 long; with right paramere rounded at apex, left paramere lobed at apex and extended further, internal sac with long sinuate rod that is asymmetrically bifurcate at apex.

**Females:** Fifth tergite with setose area on transverse rounded ridge, with median depression at apex bordered by small angulations; fifth ventrite (Fig. 16F) with setose area broadly divided by protruding glabrous area at middle. Genitalia (Fig. 16E) with medial portion strongly asymmetric, with several irregular, more heavily sclerotized areas, lobe to right with semicircular sclerotized margin.

##### Collection notes.

no collection data associated with the specimens.

##### Geographical distribution.

(Map 3): Known only from Lake and Napa Counties in the inner Coast Ranges north of the San Francisco Bay Area.

##### Comparisons and diagnostic notes.

Placed a member of the raffrayi-group. This species is most similar to *Oropodes rumseyensis* in the males sharing the broad tooth at the base of the profemora, only slightly swollen protibiae, with long apical spurs on the meso- and metatibiae, and the lamina of the third ventrite is angled at about 30°, while the female genitalia have a strongly asymmetric membranous lobe, and the fifth ventrite has the setose area divided. They differ by *Oropodes nuclere* having a more complex rod of the internal sac and the internal sac lacking a cluster of denticles in the apical portion in the males, while the females have the setose area of the fifth sternite widely divided by a bar and the apex of the fifth tergite has a median impression. For *Oropodes rumseyensis* the rod of the internal sac lacks an apical division and there is a cluster of denticles in the apical portion for the males, while the females have the setose area of the fifth ventrite divided by projections of the anterior and posterior margins that meet but do not fuse and the fifth tergite has a blunt median tubercle at the apex. The male specimen from Sequoia National Park that was placed as this species by Grigarick & Schuster (1976) is described above as the holotype of *Oropodes casson*.

#### 16. Oropodes raffrayi

(Brendel, 1894) NEW STATUS

http://species-id.net/wiki/Oropodes_raffrayi

Fig. 17

Euplectus raffrayi Brendel, 1894: 196. Type locality: California. Holotype male, // Brend/ Horn Coll H9450/ E. raffrayi Brend/ TYPE #8291 Euplectus raffrayi B. Carl Farr Moxey 29.VI.1962 // (AMNH). Listed as synonym of *Oropodes orbiceps* by Raffray (1904: 565).

##### Specimens examined.

2: holotype male, “California” (AMNH); 1 male, “30”, Schmitt Coll. (FMNH).

##### Description.

(only males known). Length 2.04. Body light yellow-brown. Eyes with about 60 facets. Antennomeres V and VII slightly larger than those adjacent, V-VIII obconical, IX narrower than X. Abdomen with carinae of first ventrite extending from inner margin of metacoxal cavities to ventrite apex.

**Figure 17.**
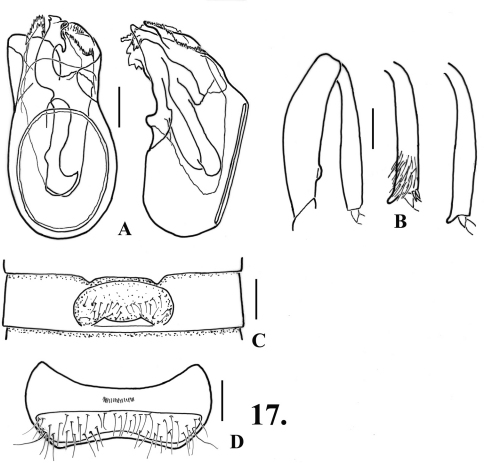
*Oropodes raffrayi* **A** Dorsal and right lateral view of male genitalia **B** Posterior view of right male profemur and protibia, mesotibia, and metatibia **C** Ventral view of male third ventrite **D** Ventral view of male sixth ventrite. Scale line equals 0.1 mm.

**Males**: Metasternum with broad median longitudinal impression through length. Legs (Fig. 17B): profemora with short, broadly truncate tubercle near base on mesal margin; protibiae slightly swollen in apical half; mesotibiae thickened, with large curving spur at mesal apex, metatibiae with shorter curved spur at mesal apex. Abdomen (Fig. 17C) with second ventrite impressed between paired teeth on apical margin, semicircular impression over most of ventrite disc, teeth 0.17 apart between centers; third ventrite 0.61 wide, impressed in medial third anterior to recurved lamina, impression with long setae, with lateral margins carinate, lamina 0.18 wide, arising near posterior margin of ventrite, apical margin broadly and shallowly emarginate, lamina strongly curved at base, with apical half horizontal; fourth-fifth ventrites flattened in medial fourth; sixth ventrite (Fig. 17D) with setose area slightly constricted in central portion, setae long compared with other species. Aedeagus (Fig. 17A) with left paramere barely longer, apex broadly rounded, right paramere obtusely angulate at lateroapical margin; internal sac with thick spine complexly toothed in apical portion, apical portion of internal sac with many small thick spines.

**Females**: unknown.

##### Collection notes.

Two males are known.

##### Geographical distribution.

The only distributional data available is the broad statement of “California” for the holotype, while the second specimen lacks any collection data.

##### Comparisons and diagnostic notes.

The species upon which the raffrayi-group is based. The males share a number of features with *Oropodes nuclere* and *Oropodes rumseyensis*, species of the Coast Range north of the San Francisco Bay area, such as the broad basal tubercle of the profemora, the long apical spurs of the meso- and metatibiae, and the large rod of the internal sac which has a number of apical teeth. The horizontal lamina of the third ventrite, and the more complexly toothed apex of the rod in the internal sac will separate *Oropodes raffrayi* from these two species. Both *Oropodes raffrayi* and *Oropodes rumseyensis* are the only two species that have distinct denticles of the internal sac in its apical portion.

The distribution of this species is still unknown, but the discussion above suggests that it may be a species of northern California.

#### 17. Oropodes rumseyensis

Grigarick & Schuster, 1976

http://species-id.net/wiki/Oropodes_rumseyensis

Fig. 18 Map 3

Oropodes rumseyensis Grigarick & Schuster, 1976, 100; Chandler 1997: 15. Type locality: California, Yolo County, 3 mi N Rumsey. Holotype male (UCDC). Grigarick & Schuster, 1980: pl. 29.

##### Specimens examined.

15: CALIFORNIA: **Marin County:** 2 km S Olema, 20 m, N38 00.5’, W122 46’, V-9/11-2003, S.B. Peck, mixed ravine forest, FIT (DSC). **Napa County:** 2 mi N St. Helena, White’s Cove entrance, IV-26/VIII-19-1981, R.L. Aalbu, antifreeze pit trap (CSCA, DSC). Napa, II-3-1959, R.O. Schuster (UCDC). **Solano County:** Mix Canyon, III-12-1960 (UCDC). **Tehama County**: Patton Mill, VIII-30-1960, R.O. Schuster (UCDC). **Yolo County:** 3 mi N Rumsey, VII-29-1959, R.O. Schuster & L.M Smith (DSC, 1 female paratype; UCDC, 1 male, 9 female paratypes).

##### Description.

Length 1.84-1.94 mm. Body light orange-brown. Eyes of both sexes with about 60 facets. Antennomeres V and VII slightly larger than those adjacent, V-VIII obconical, IX narrower than X. Abdomen with carinae of first ventrite extending from inner margin of metacoxal cavities to ventrite apex.

**Figure 18.**
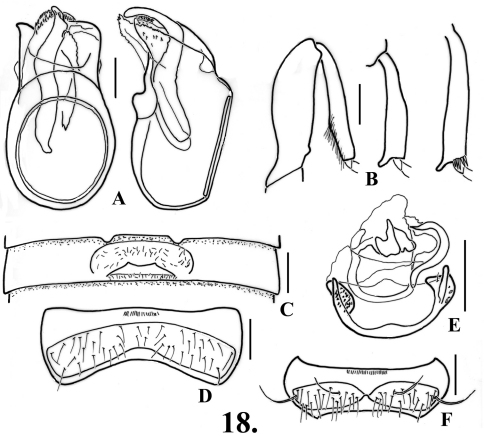
*Oropodes rumseyensis* **A** Dorsal and right lateral view of male genitalia **B** Posterior view of right male profemur and protibia, mesotibia, and metatibia **C** Ventral view of male third ventrite **D** Ventral view of male sixth ventrite **E** Dorsal view of female genitalia **F** Dorsal view of female fifth ventrite. Scale line equals 0.1 mm.

**Males:** Metasternum with median longitudinal impression. Legs (Fig. 18B): profemora with small blunt tubercle near base on mesal margin; protibiae slightly angularly swollen on mesal margin past middle; meso- and metatibiae with prominent apical spurs on mesal margin, spurs subequal in size. Abdomen (Fig. 18C) with second ventrite slightly depressed in medial fourth from middle to apex, with pair of widely separated rounded teeth on apical margin, teeth 0.16 apart between centers; third ventrite 0.62 wide, impressed in medial third, lamina 0.14 wide, arising near posterior margin of ventrite, angled at about 30°, apex of lamina shallowly emarginate; fourth-fifth ventrites slightly impressed in medial third; sixth ventrite (Fig. 18D) slightly impressed in medial fourth, with setose area slightly constricted at middle. Aedeagus (Fig. 18A) 0.48 long; left paramere broadly subtruncate at apex, right paramere with apex pointed obliquely laterally; internal sac with long sinuate rod, apex simple.

**Females:** Fifth tergite with blunt medial tubercle at apex, setose area transversely convex and bulging. Fifth ventrite (Fig. 18F) with setose area completely constricted at middle, margins meeting but not fused. Genitalia (Fig. 18E) strongly asymmetric, with large rounded lobe on right margin edged by arcuate sclerite.

##### Collection notes.

Taken from dry or scrub forests at low elevations.

##### Geographical distribution.

(Map 3): Most records are from the inner Coast Ranges extending from the San Francisco Bay Area north to Tehama County, with one specimen taken at the coast in Marin County.

##### Comparisons and diagnostic notes.

Placed as a member of the raffrayi-group. This species is most similar to *Oropodes nuclere* in the males sharing the broad tooth at the base of the profemora, only slightly swollen protibiae, with long apical spurs on the meso- and metatibiae, and the lamina of the third ventrite is angled at about 30°, while the female genitalia have a strongly asymmetric membranous lobe, and the fifth ventrite has the setose area divided. They differ by the males of *Oropodes rumseyensis* having an apically undivided rod in the internal sac and there is a cluster of denticles in the apical portion, while the females have the setose area of the fifth ventrite divided by projections of the anterior and posterior margins that meet but do not fuse and there is a blunt median tubercle at the apex of the fifth tergite. The males of *Oropodes nuclere* have an apically divided rod in the internal sac and the internal sac lacking a cluster of denticles in the apical portion, while the females have the setose area of the fifth sternite widely divided by a bar.

#### 18. Oropodes tongva

Chandler & Caterino sp. n.

urn:lsid:zoobank.org:act:074288AE-43FE-46E2-9FD7-EF5CD526D1EF

http://species-id.net/wiki/Oropodes_tongva

Fig. 19 26 Map 3

##### Specimens examined.

16: HOLOTYPE male: California, Los Angeles Co., Angeles National Forest, SDEF [San Dimas Experimental Forest], Tanbark Flat, 34.2084°N, 117.7637°W, IV-15/29-2007, Caterino & Chatzimanolis, FIT (SBMN; CBP0064383). PARATYPES: same data except, IV-15/29-2007 (2M, 8F), V-13/28-2007 (4F), VI-23/VII-1-2007 (1M) (DSC, CASC, LACM, SBMN).

##### Description.

Length 2.04-2.16. Body orange-brown. Eyes with 50-65 facets. Antennomeres V and VII slightly larger than those adjacent, V-VIII obconical, IX narrower than X. Abdomen with carinae of first ventrite extending from posteromesal margins of metacoxal cavities to ventrite apex.

**Figure 19.**
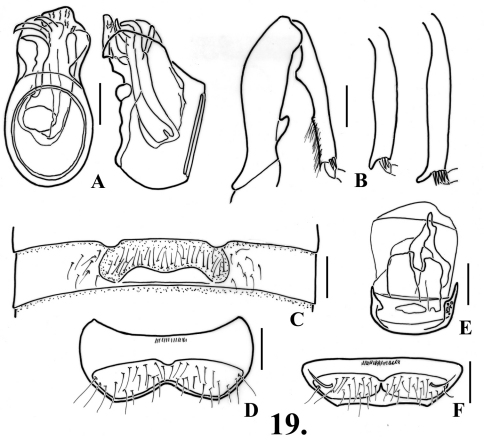
*Oropodes tongva* **A** Dorsal and right lateral view of male genitalia **B** Posterior view of right male profemur and protibia, mesotibia, and metatibia **C** Ventral view of male third ventrite **D** Ventral view of male sixth ventrite **E** Dorsal view of female genitalia **F** Dorsal view of female fifth ventrite. Scale line equals 0.1 mm.

**Males**: Metasternum with median longitudinal sulcus. Legs (Fig. 19B): profemora with large oblique ventral tooth near base, protibiae with large subtruncate tubercle near base on mesal margin, margin excavate to smaller tubercle past midpoint, protibiae narrowing slightly to apex from that point; mesotibiae with large straight apical spur on mesal margin, metatibiae with large curved apical spur on mesal margin. Abdomen (Fig. 19C) with second ventrite gently concave in apical third to form semicircular impression, setae sparse in impression, lateral margins of impression arising just lateral to pair of apically rounded teeth, teeth 0.22 apart at their centers; third ventrite 0.6 wide, with prominent transverse recurved lamina arising at basal margin, lamina 0.18 wide, angled at about 40°, anterior margin broadly convex, transverse impression anterior to tubercle with lateral margins carinate, with dense thickened setae in impression; fourth ventrite gently concave in medial third; fifth ventrite gently concave in medial fourth; sixth ventrite (Fig. 19D) flat in medial fourth, setose area constricted at middle to about half of lateral length. Aedeagus (Fig. 19A) 0.40 long, left paramere subtruncate and longer, right paramere broadly rounded at apex, with two laterally curved spines at apex in internal sac.

**Females**: Metasternum with median longitudinal sulcus present as in male. Fifth tergite with setose area convex. Sixth ventrite (Fig. 19F) with setose area divided by narrow median bar, or nearly closed by median carina. Female genitalia (Fig. 19E) with membranous lobe symmetrical, but sclerites within lobe asymmetrical, with two broad subtruncate and lightly sclerotized plates in membranous lobe.

##### Collection notes.

All specimens originated from a single site, taken by flight intercept trap from April to July. The collection site was located in a small pocket of live oak (*Quercus agrifolia*) woodland surrounded by mid-elevation chaparral.

##### Geographical distribution.

(Map 3): The series was taken from the San Gabriel Mountains, on the northern margin of the Los Angeles Basin.

##### Comparisons and diagnostic notes.

Placed as a member of the raffrayi-group, together with six other species. This species is unique in the large, oblique, basal profemoral tooth, strong basal angulation of the protibiae, and the long apical spurs of the meso- and metatibiae of the males, while the females have the setose area of the fifth ventrite slightly interrupted at the middle. This is the only species that has the median longitudinal sulcus of the metasternum present in both sexes.

##### Etymology.

The specific epithet, treated as a Latin singular noun in apposition, nominative case, is based on the tribal name of the Tongva Indians, who originally lived in the area where the specimens of this species were collected.

**Figures 20–26.**
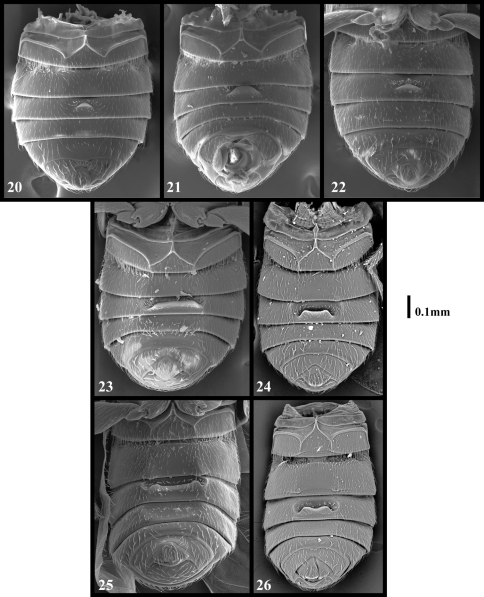
Scanning electron micrographs, ventral view of male abdomens. Scale line equals 0.1 mm **20** *Oropodes arcaps* **21** *Oropodes ishii* **22** *Oropodes yollabolly* **23** *Oropodes dybasi* **24** *Oropodes orbiceps* **25** *Oropodes chumash* **26** *Oropodes tongva*. Scale line equals 0.1 mm.

## Supplementary Material

XML Treatment for Oropodes

XML Treatment for Oropodes arcaps

XML Treatment for Oropodes dybasi

XML Treatment for Oropodes ishii

XML Treatment for Oropodes yollabolly

XML Treatment for Oropodes orbiceps

XML Treatment for Oropodes serrano

XML Treatment for Oropodes tataviam

XML Treatment for Oropodes tipai

XML Treatment for Oropodes aalbui

XML Treatment for Oropodes bellorum

XML Treatment for Oropodes casson

XML Treatment for Oropodes chumash

XML Treatment for Oropodes esselen

XML Treatment for Oropodes hardyi

XML Treatment for Oropodes nuclere

XML Treatment for Oropodes raffrayi

XML Treatment for Oropodes rumseyensis

XML Treatment for Oropodes tongva
